# Investigating the Neuroprotective and Cognitive-Enhancing Effects of *Bacopa monnieri*: A Systematic Review Focused on Inflammation, Oxidative Stress, Mitochondrial Dysfunction, and Apoptosis

**DOI:** 10.3390/antiox13040393

**Published:** 2024-03-25

**Authors:** Luiz José Valotto Neto, Matheus Reverete de Araujo, Renato Cesar Moretti Junior, Nathalia Mendes Machado, Rakesh Kumar Joshi, Daiane dos Santos Buglio, Caroline Barbalho Lamas, Rosa Direito, Lucas Fornari Laurindo, Masaru Tanaka, Sandra Maria Barbalho

**Affiliations:** 1 Department of Biochemistry and Pharmacology, School of Medicine, University of Marília (UNIMAR), Marília 17525-902, SP, Brazil; valottonetoluizjose@gmail.com (L.J.V.N.); reveretem@gmail.com (M.R.d.A.); renatocesar_moretti@outlook.com (R.C.M.J.); nathalia.m.machado@outlook.com (N.M.M.); daiane_santos_buglio@outlook.com (D.d.S.B.); 2 Department of Education, Government of Uttarakhand, Nainital 263001, India; raakeshjoshi@rediffmail.com; 3 Department of Gerontology, School of Gerontology, Federal University of São Carlos (UFSCar), São Carlos 13565-905, SP, Brazil; carolinelamas@estudante.ufscar.br; 4 Laboratory of Systems Integration Pharmacology, Clinical & Regulatory Science, Research Institute for Medicines (iMed.ULisboa), Faculty of Pharmacy de Farmácia, University of Lisboa, 1649-003 Lisbon, Portugal; rdireito@ff.ulisboa.pt; 5 Faculty of Pharmacy, University of Lisboa, Av. Prof. Gama Pinto, 1649-003 Lisbon, Portugal; 6 Department of Biochemistry and Pharmacology, School of Medicine, Medical School of Marilia (FAMEMA), Marília 17519-030, SP, Brazil; lucas_fornari_laurindo@outlook.com; 7 Danube Neuroscience Research Laboratory, HUN-REN-SZTE Neuroscience Research Group, Hungarian Research Network, University of Szeged (HUN-REN-SZTE), Tisza Lajos krt. 113, H-6725 Szeged, Hungary; 8 Postgraduate Program in Structural and Functional Interactions in Rehabilitation, School of Medicine, Universidade de Marília (UNIMAR), Marília 17525-902, SP, Brazil; 9 Department of Biochemistry and Nutrition, School of Food and Technology of Marília (FATEC), Marília 17500-000, SP, Brazil

**Keywords:** *Bacopa monnieri*, neurodegenerative diseases, Alzheimer’s disease, cognition, oxidative stress, inflammation, mitochondrial dysfunction

## Abstract

The aging of the global population has increased the prevalence of neurodegenerative conditions. *Bacopa monnieri* (BM), an herb with active compounds, such as bacosides A and B, betulinic acid, loliolide, asiatic acid, and quercetin, demonstrates the potential for brain health. Limited research has been conducted on the therapeutic applications of BM in neurodegenerative conditions. This systematic review aims to project BM’s beneficial role in brain disorders. BM has anti-apoptotic and antioxidant actions and can repair damaged neurons, stimulate kinase activity, restore synaptic function, improve nerve transmission, and increase neuroprotection. The included twenty-two clinical trials demonstrated that BM can reduce Nuclear Factor-κB phosphorylation, improve emotional function, cognitive functions, anhedonia, hyperactivity, sleep routine, depression, attention deficit, learning problems, memory retention, impulsivity, and psychiatric problems. Moreover, BM can reduce the levels of pro-inflammatory biomarkers and oxidative stress. Here, we highlight that BM provides notable therapeutic benefits and can serve as a complementary approach for the care of patients with neurodegenerative conditions associated with brain disorders. This review adds to the growing interest in natural products and their potential therapeutic applications by improving our understanding of the mechanisms underlying cognitive function and neurodegeneration and informing the development of new therapeutic strategies for neurodegenerative diseases.

## 1. Introduction

The average age of the world’s population has been rising sharply since the 1970s, and this trend is expected to continue throughout the 21st century. Gradual changes in cell metabolism characterize biological aging. This natural and irreversible process can occur in a typical or pathological manner [1,2]. With the rapid growth of adults aged 65 and over, many metabolic and neuronal conditions such as type 2 diabetes mellitus (DM2), musculoskeletal diseases, cardiovascular diseases, atherosclerosis, hypertension, frailty, and neurological diseases can be observed. Allied to that, physical frailty is a clinical state with increased susceptibility to stressors due to reduced accounts in multiple physiological systems, resulting in functional decline and increased mortality [3,4,5,6,7,8,9,10,11,12,13,14].

Cognitive decline is a common phenomenon observed in an aging population. Dementia is one of the most feared neurological disorders, and the number of people with dementia is rapidly increasing. Alzheimer’s disease, a neurodegenerative condition defined by the high accumulation of neurofibrillary tangles and amyloid plaques in the brain, and Parkinson’s disease, characterized mainly by tremor, rigidity, bradykinesia, and postural instability, are conditions that the elderly face over time [15,16,17,18,19,20].

To maintain or improve brain health or slow and reverse dementia, nearly a quarter of adults currently use some dietary supplement, including vitamins, minerals, and amino acids [17,21,22,23]. Novel therapeutic opportunities for neurodegenerative diseases include strategies for enhancing cognitive function and reducing amyloid deposition, presenting new avenues for the treatment of these conditions [24,25,26]. Medicinal plants and herbal remedies are also becoming relevant as complementary interventions, providing a valuable source for drug development for neurodegenerative diseases [27,28,29]. This global use is driven by a strong interest in younger and older generations to improve cognitive performance and achieve healthy aging [30,31,32,33,34].

**Figure 1:**
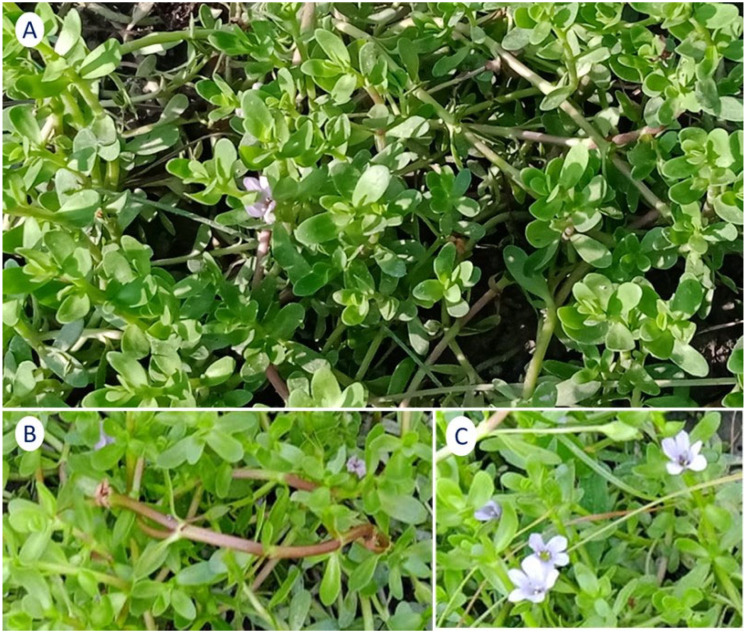
BM plant. (**A**) whole plant; (**B**) stem detail; (**C**) flower detail.

Many studies have considered *Bacopa monnieri* L. (BM) to prevent or treat neurodegenerative conditions [35,36]. This plant is known as brahmi, water hyssop, *Herpestis monniera*, or “medhya rasayanas” in Ayurveda (meaning brain tonic). It is a small creeping perennial herb with numerous branches, small oblong leaves, and light purple flowers (Figure 1) growing in wetlands. It has been used in many parts of the world as a medicine, particularly for treating various neurological disorders such as improving memory, thinking skills, insomnia, seizures, and anxiety [37,38,39,40,41]. In a review performed by Fatima et al. [40], the authors studied the molecular pathways in therapeutic interventions of BM in several metabolic disorders and also in Alzheimer’s and Parkinson´s diseases. They suggested that its use has promising effects regarding neuronal impairments. Other authors have shown BM constituents’ effects as benefits in dementia, Alzheimer´s disease, and other brain disorders [42,43,44].

Recently, this plant has been widely described for its pharmacologically active phytoconstituents, such as bacopasaponin C, bacosides A and B, bacopasides I and II, loliolide, betulinic acid, asiatic acid, ebelin lactone, and quercetin. BM mechanism of action in brain diseases may be related to its ability to modulate neurotransmission, neurogenesis, neuronal plasticity, intracellular signaling, epigenetics, cerebral blood flow, energy metabolism, protein folding, endoplasmic reticulum stress, neuroendocrine system, and apoptosis [44,45,46,47]. BM is also known for its antioxidant, anti-inflammatory, and anti-hepatotoxic functions. Antioxidant and anti-inflammatory activities are of fundamental importance in avoiding neurodegenerative processes [48,49,50,51,52].

One of the current challenges is the limited understanding of therapeutic applications of BM in neurodegenerative conditions. Although there is evidence that BM has the potential to improve cognitive disorders and prevent oxidative damage, its mechanisms against neurodegenerative diseases are yet to be fully understood. Furthermore, clinical evidence for BM’s efficacy and safety of BM in neurodegenerative diseases is limited and inconsistent, and the molecular mechanisms by which BM exerts its neuroprotective effects are not fully understood. Considering the intimate relationship between population aging and organic dysfunctions such as degenerative diseases, this systematic review was built to underline the question: ‘’Can BM L. benefit neurodegenerative diseases?’’.

## 2. Literature Search Strategy and Study Selection

This systematic review aimed to investigate the potential benefits of BM L. for neurodegenerative diseases. Only clinical trials published in English were considered, sourced from COCHRANE, EMBASE, and MEDLINE–PubMed databases. The search utilized mesh terms including BM L. or brahmi, along with antioxidant, anti-inflammatory, anti-apoptotic, aging, neurodegenerative disease, Alzheimer’s disease, Parkinson’s disease, memory, memory scale, cognition, and cognitive impairment. These terms guided the identification of studies examining BM’s effects on neurodegenerative diseases associated with aging. Study identification and inclusion were carried out by S.M.B. and L.F.L., with conflicts resolved by a third judge, M.T. Inclusion criteria were limited to human interventional studies, while exclusion criteria comprised studies not in English, editorials, conferences, letters to editors, reviews, poster presentations, and case reports. The search for clinical trials had no temporal restriction. Data extraction followed the PICO (Population, Intervention, Comparison, and Outcomes) format, and study selection adhered to PRISMA (Preferred Reporting Items for Systematic Reviews and Meta-Analyses) guidelines [53,54]. The risk of bias in selected trials was evaluated using the Cochrane Handbook for systematic reviews of interventions [55].

## 3. Overview of the Included Studies and Result Findings

**Figure 2:**
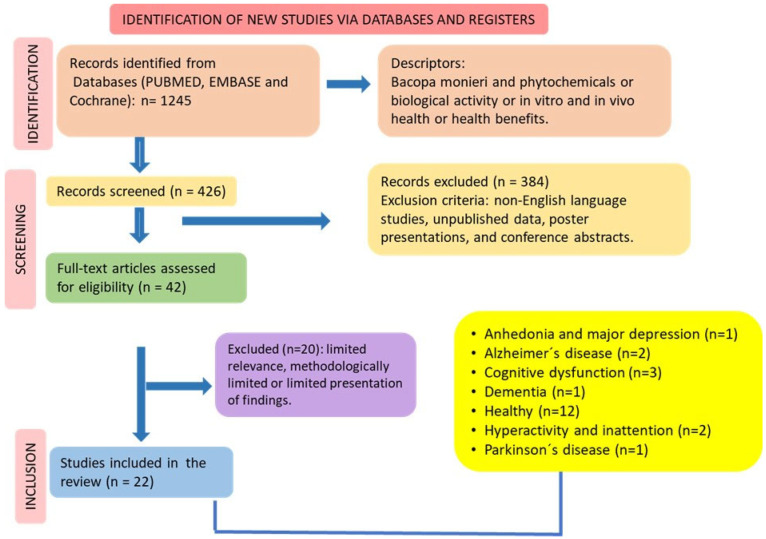
Flow diagram showing the study selection (PRISMA Guidelines) [53,54].

After applying the inclusion/exclusion criteria, we selected twenty-two clinical trials (Table 1) related to using BM in the following conditions: anhedonia and major depression, Alzheimer’s disease, Parkinson´s disease, dementia, hyperactivity, and inattention. Twelve studies investigated the use of the plant in healthy patients (Figure 2). Seventeen studies compared the results with placebo. The dosages varied from 160 to 640 mg, and the treatment period varied from four weeks to six months.

The main adverse events, when reported, were nausea, stomach pain, headache, dizziness, anxiety, and diarrhea. Some studies used formulations such as BacoMind, Keen-Mind, and CDRI-08.

Three studies investigated the effects of consumption of BM associated with other compounds. One mix included a total formulation of 500 mg of BM, *Hippophae rhamnoides*, and *Dioscorea bulbifera* [66]. Another study used the combination of BM (320 mg), *Crocus sativus* 30 mg, biotine 450 mcg, vitamin B6 (9.5 mg), folic acid 400 mcg, vitamin B12 (33 mcg), vitamin D (25 mcg), L-theanine 100 mg, and cupper (2 mg) [64]. The other combination was composed of BM, vitamin B12, astaxanthin, and lycopene.

Overall, the results showed that the use of BM (alone or combined with other compounds) could increase Cyclic AMP response element-binding protein (CREB) phosphorylation and reduce Nuclear Factor-κB (NF-κB) phosphorylation, improve emotional function, cognitive function, cognitive flexibility, anhedonia, hyperactivity, executive functioning, interpersonal problems, sleep routine, depression, attention-deficit, learning problems, learning rate, memory retention, memory accuracy, impulsivity, and psychiatric problems. BM leaf extract have memory enhancing effect by augmenting 5-hydroxytryptamine (5HT) metabolism, transportation and further activation of 5-HT-3A receptor during hippocampus-dependent learning. BM can modulate microRNA 124-CREB pathway to improve synaptic plasticity, improving cognition and memory [78].

The use of the extract also improved the Mini-Mental State Examination (MMSE) and Perceived Stress Questionnaire (PSQ) Index. Moreover, BM can reduce the levels of pro-inflammatory biomarkers and oxidative stress.

In general, the clinical trials included both sexes. Two studies were performed with children (6–14 years old) [58,67] and the others with adults and elderly participants.

The main biases for the included studies are small-sized samples, the lack of blindness, and the lack of comparison with a placebo group (Table 2). These biases can interfere with the results from statistics analysis and reduce the power of evidence for using BM in brain conditions.

## 4. Understanding the Neuroprotective and Cognitive-Enhancing Effects of *Bacopa monnieri*: Focus on Inflammation, Oxidative Stress, and Mitochondrial Dysfunction

### 4.1. Bacopa monnieri (BM)

BM is an important medicinal herb with a rich historical and religious history of over 1400 years. It has high medicinal value and ranks second on the list of essential Indian foods. Belonging to the Plantaginaceae family, BM can grow in East Asia in rice plantations and the United States of America. The entire plant is used as a brain and nervous system booster to treat psychological disorders and skin problems. The leaf juice is applied to inflamed areas and serves as a lotion to treat rheumatism [40,59,79,80].

Herbal supplements have long played key roles in treating neuronal and pathological disorders with no or limited side effects. The components present in BM are alkaloid brahmine, nicotinine, herpestine, bacosides A and B, saponins A, B, and C, triterpenoid saponins, stigmastanol, β-sitosterol, betulinic acid, D-mannitol, stigmasterol, α-alanine, aspartic acid, glutamic acid, and serine and pseudojubogenin glycoside. By now, the main potential effects on cognition and memory are related bacoside A, bacoside B, alkaloids, and flavonoids. The actions include increasing neurotransmission, enhancing synaptic activity, and repairing damaged neurons by upregulating neuronal synthesis and kinase activity [81,82,83]. The bacosides play essential actions in neuronal health due to their anti-inflammatory, antioxidant, antiapoptotic, antidepressant, antiapoptotic, reduction of mitochondrial impairment, reduction of endoplasmatic reticulum stress, improvement of neurogenesis, cerebral blood flow, energetic metabolism, neuroprotection, and several other actions. There are at least 12 bacosides analogs, known as bacopasides I–XII. They have the ability to avoid cytotoxicity and DNA damage in neurons related to Alzheimer’s disease. They can also repair the impaired neurons due to the stimulation of kinase activity and neuronal synthesis. They can inhibit the production biomarkers such as TNF-α, IL-6, IL-17a, and CCL5. Other described action of bacosides refers to the prevention of the leakage of Creatine Kinase from the respective brain and cardiac tissues. Further, they induce the phosphorylation of AMP-activated protein kinase (AMPK) in many tissues. Bacoside A and bacoside B are associated with most nootropic effects of neuropharmacological actions; however, bacoside A is pharmacologically more active than B. Some bacosides derivatives are named Bacogenin A1–A5, and ebelin lactone (bacogenin A4) are the major compounds. Bacopasapin A-H are jujubogenin and pseudojujubogenin derivatives, and bacopasaponin C accounts for 0.3–0.6% of BM ethanolic extracts. Cucurbitacin, bacitracin A–D, and cucurbitacin E play several roles in plants and animals and are also present in BM extracts, as well as betulinic acid [40,45,84].

BM water or alcoholic extracts are also recognized by exhibiting dammarene type triterpenoid saponins (with jujubogenin or pseudo-jujubogenin as aglycones), bacopaside I–III, III–V, and bacosides A1–A3, and bacopasaponins A–G. However, as pointed out before, the nootropic actions are mainly related to the presence of bacoside A and B. Furthermore, three triterpinoid saponins are also found in the extracts: bacoside A1 [3-*O*[α-larabinofuranosyl (1/3) α-L-arabinopyranosyl]jujubogenin], bacoside A2 [3β-[*O*α-L- arabinofuranosyl(1/6)-*O*-[α-L-arabinopyranosyl(1/5)]-*O*-α-Dglucofuranosyl) oxy]pseudojujubogenin], and bacoside A3 [3-β-[*O*-β-d-glucopyranosyl(1/3)-*O*-[α-L-arabinofuranosy (1/2)]-*O*-β-d-glucopyranosyl)oxy]jujubogenin] [44,47,83,85,86,87]. Table 3 brings the main compounds present in BM and their biological effects.

### 4.2. BM and Inflammation

Inflammation is one of the most common immune response mechanisms against infections, diseases (cardiovascular, metabolic, neurodegenerative, and cancer), and tissue injuries [112]. It is notable that systemic inflammation increases with age, evidenced by higher circulating levels of pro-inflammatory cytokines, Interleukin-1β (IL-1 β), Interleukin-6 (IL-6), Tumor necrosis factor alpha (TNF-α), and immune dysregulation [113]. Neuroinflammation is a crucial defense mechanism that protects the brain by removing or inhibiting various pathogens. This response can have beneficial effects by promoting tissue repair and removing cellular debris. However, when prolonged, it is harmful and inhibits regeneration [114,115,116]. Inflammatory stimulation may persist due to endogenous (e.g., genetic mutation and protein aggregation) or environmental (e.g., infection, trauma, and drugs) factors involving microglia and astrocytes, leading to neurodegenerative diseases [117,118]. Diseases that induce progressive cognitive and functional impairment, such as Alzheimer’s disease, Parkinson’s disease, among other related dementias, are among the main contributors to disability and mortality. After the age of 65, the incidence of such pathologies increases exponentially every five years, and after the age of 90, approximately one in three adults meets the criteria for dementia [119,120,121,122,123].

Several products from plants are used to treat neuroinflammation, including BM. Administration of bacoside A, one of the main components of BM, significantly inhibits TNF-α and many inflammatory enzymes, Metalloproteinase 3 (MMP-3) and caspases 1 and 3, and the release of IL-6 from microglial cells, which is the main modulator of neuroinflammation in the Central Nervous System (CNS) [47,124]. These activated microglia produce pro-inflammatory proteins, which lead to prolonged neuroinflammation and cause critical processes associated with neurodegenerative diseases [125,126]. In addition to Bacosides, indolic compounds and phenolic acids are also responsible for the anti-inflammatory action, reducing cyclooxygenase 2 (COX-2) and Prostaglandin C (cPGES) expression [41,95,127,128].

A study evaluated the taxonomic verification of the four wild populations of BM, the chemical content of their pigments and phenols and provided an analysis of their potential bioactivity. The chromatographic profiling revealed 21 compounds including 12 chlorophylls and 9 carotenoids, in which lutein and β-carotene were the major ones. The total phenolic content ranged from 54.8 ± 5.8 to 70.3 ± 2.2 mcg of gallic acid equivalents/mg. The scavenging activity of BM extracts against the free radical DPPH exhibited IC_50_ values ranging from 130.6 ± 3.0 to 249.9 ± 12.1 mcg of dry extract per milliliter. Furthermore, the most effective anti-inflammatory extract was from a soil-based plant from Jalisco, reduced from nitric oxide in a RAW 264.7 culture medium, with an IC_50_ value of 134 mcg of dry extract/mL [129].

Also, it has been demonstrated that another constituent of BM, flavonoids, which are luteolin, apigenin, wogonin, quercetin, oroxidindin, and luteolin7-glucoside, suppress the expression of pro-inflammatory mediators, such as the Nuclear Factor-κB (NFκB) cascade, have vasodilatory activity, improve vascular endothelial function, protect cells against insulin resistance, regulate proliferation, inhibit activation of the complement system and suppress neuroinflammation by reducing the release of cytokines [130,131,132].

The effects of BM ethanolic extract were investigated in Alzheimer’s disease conditions resulting from the injection of amyloid-β_42_ (Aβ_42_) fibril into Wistar rat’s hippocampus. The results showed that the extract orally administered for four weeks ameliorated the animals’ cognitive impairment and explorative behavior. Furthermore, the use of the extract reduced proinflammatory cytokines, oxidative stress markers, and cholinesterase activity. The authors also observed an increase in the expression of neurotrophic factors, restoration of Bcl-2-associated X protein (Bax)/B-cell lymphoma 2 (Bcl-2) imbalance, and prevention of neurodegeneration in hippocampal neurons. The use of the extract removed amyloid plaques and normalized Aβ_42_-induced increase in phospho-tau. The effects produced by BM in rats are due to its interaction with glycogen synthase kinase (GSK-3β) and restoring Wnt/β-catenin signaling pathways [35].

A pilot study showed that BM phytocompounds such as quercetin can exhibit a relevant anti-inflammatory action. The results showed the interaction with inducible Nitric Oxide synthase (iNOS), Cyclooxygenase-2 (COX-2), Lipoxygenase (LOX), C*-C chemokine receptor type 1* (CCR1), MMP9, and signal transducers and activators of transcription (STAT3) genes [133]. Constantini et al. [134] evaluated the anti-inflammatory effects of BM extract in neuroinflammation in vitro model (neuronal SH-SY5Y cells that underwent stimulation with Interferon-γ (INF-γ) and TNF-α regarding toxicity, cell viability, and cytokine gene expression. Their results showed that BM extract can inhibit Okadaic acid-induced cytotoxicity in the studied cells. The use of the extract also reduced the levels of COX-2, IL-1β, IL-6, and iNOS. The results of this study indicate that BM can be used in the treatment of neuroinflammation.

Another investigation showed that BM can reduce acute pain and inflammation by inhibiting COX-2 and reducing prostanoid mediators arbitrated by this enzyme. This study also hypothesizes that the potential phytocompounds identified from BM can be employed to regulate signaling pathways associated with target genes, which could lead to treating inflammation-responsive diseases. As expected, the results obtained in vitro revealed that the identified potential phytocompound effectively inhibits the gene expression responsible for inflammation, such as COX2 and iNOS, in LPS-induced RAW264.7 cells [133].

Some authors found that chemotherapy-induced cognitive impairment is a new clinical condition characterized by deficits in memory, learning, and motor function. Oxidative stress and inflammatory processes are potential factors that lead to the adverse effects of chemotherapy on the brain. Inhibition of soluble epoxide hydrolase has proven effective in neuroinflammation and reversing memory impairment. Treatment with the dual inhibitor of COX and epoxide hydrolase (PTUPB) or herbal extracts inhibiting Soluble Epoxide Hydrolase (sHE) preserved spatial memory by ameliorating oxidative stress and inflammation. BM performed better than expected in preserving spatial memory by ameliorating oxidative stress and inflammation [135].

Other authors infused acetic acid intrarectally to induce ulceration in mice, resulting in severe inflammation of the colon. Thus, with the use of BM extract (20 mg/kg and 40 mg/kg, orally) and saponin-rich fraction (5 mg/kg and 10 mg/kg; orally) for seven days, i.e., two days before and five days after acetic acid infusion, a significant reduction in inflammation was observed [136].

A study evaluated the neuroprotective impact of a standardized extract from BM (BacoMind) against the valproic acid-induced model of autism spectrum disorder (ASD) in rats. The results of the in vivo study indicated that BM at 80 mg/kg improved abnormal behavioral paradigms such as social deficits, repetitive behavior, and impairments in learning, memory, and motor coordination exhibited by the valproic acid model of ASD. The authors also report that BM has significant antioxidant (increasing Glutathione peroxidase (GPx), Superoxide Dismutase (SOD), and catalase and decreasing Malonaldehyde (MDA) levels) and anti-inflammatory (decreasing IL-1β, 6, and TNF-α) properties [137].

Other authors investigated the inhibitory capacity of BM on the release of pro-inflammatory cytokines by microglial cells. Both Bacoside A and tea, infusion, and alkaloid extracts of BM demonstrated significant inhibition in releasing TNF-α and IL-6 by activated N9 microglial cells in vitro. Furthermore, BM compounds exhibited efficacy in inhibiting caspases 1 and 3 and matrix metalloproteinase-3, as assessed in the cell-free assay. Therefore, Bacopa has the potential to attenuate inflammatory processes in the CNS and emerges as a promising source for the development of new therapeutic approaches targeting various neurological disorders [138].

The potential preventive capacity of BM supplementation in relation to colchicine-caused inflammation and Aβ production was also evaluated. Dementia was induced by a single intracerebroventricular administration of colchicine (15 mcg/5 μL), while BM extract was administered orally (50 mg/kg body weight, daily) for 15 days. An increase in the expression of pro-inflammatory cytokines (IL-6, TNF-α) and Monocyte chemoattractant protein-1 (MCP-1) was observed in brain regions. Furthermore, the expression of COX-2 and iNOS showed a significant increase in the brain areas of animals subjected to colchicine administration. Concomitantly, BACE-1 enzyme activity increased in animals treated with colchicine, increasing Aβ production. In contrast, BM supplementation has demonstrated the ability to enhance cognitive functions by suppressing Aβ formation through a reduction in beta-site amyloid precursor protein cleaving enzyme 1 (BACE-1) activity. The existence of inflammation and oxidative stress markers in the brain areas of animals supplemented with BM was attenuated. Taken together, these findings indicate that BM is capable of reversing colchicine-induced dementia through its anti-inflammatory and antioxidant properties, which suggests that this therapeutic intervention may be associated with the depletion in the progression of Alzheimer’s disease [139].

The study performed by Alharbi [140] investigated the effects of BM phytocompounds and the possible inhibitory results in schizophrenia biomarkers by a pathway analysis and protein-protein interactions (PPI) using a system biology methodology (UniProt IDs from the Reactome database were used). After implementing the computational methodology (IPP analysis), the results showed that compounds from BM can be applied to developing and treating neuronal disorders. Other authors also used a computational approach to investigate the effects of BM in understanding the molecular interaction of the phytocompounds against neurotrophic factors. The study applied molecular dynamic simulation and molecular docking to investigate the interactions of bio compounds with neurotrophins and showed that Benzene propanoic acid, vitamin E, stigmasterol, and nonacosane have an excellent binding affinity with their neurotrophins. These results indicate that BM compounds can replace synthetic drugs against neurodegenerative disorders [141].

BM aqueous extract was also used in a *Drosophila melanogaster* Aβ42 expressing model by using a quantitative proteomics investigation. The results exhibited that about ninety percent of differentially expressed proteins from Aβ42 could be restored to the original pattern after treatment. These findings are due to restoring proteins related to mitochondrial dynamics, cycle re-entry, and apoptosis. The neuroprotective actions were also related to the protective role regarding behavioral impairments promoted by Aβ42 toxicity [142].

An experimental study with Wistar rats compared the effects of reserpine, citalopram (both are drugs commonly used in the treatment of depression), and BM extract. The levels of dopamine, serotonin, and norepinephrine were evaluated, as well as the levels of FAS, Bcl-2, BDNF, and MCP-1, and the results showed that BM is capable of improving depression comparable to reserpine and citalopram in the studied model [128].

**Figure 3:**
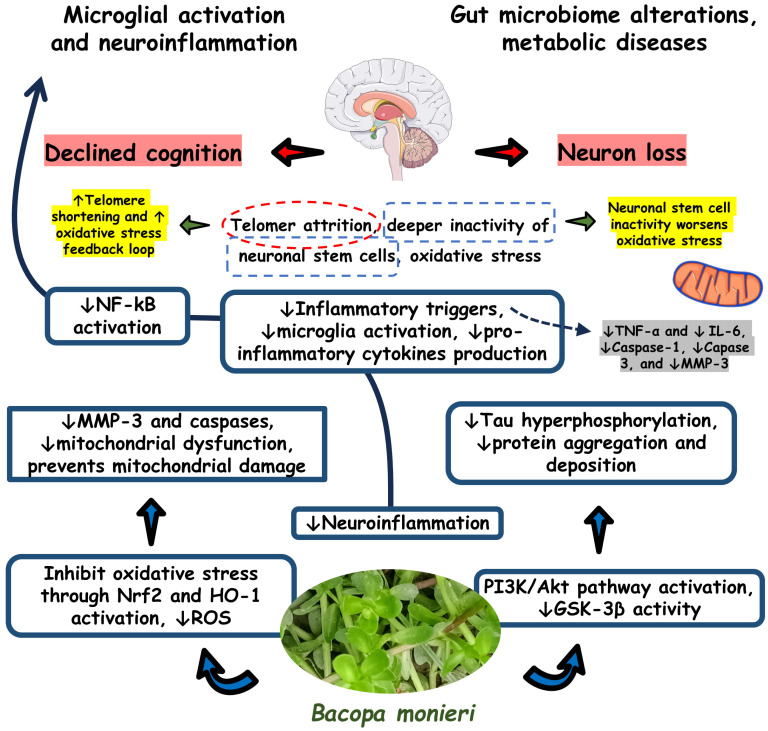
Anti-inflammatory effects of *B. monieri* on declined cognition and neuron loss. GSK-3β, glycogen synthase kinase; MMP: metalloproteinase; NF-kB, Nuclear Factor Kappa Beta; Nrf2: Nuclear factor erythroid 2-related factor 2. ↑: increase; ↓: decrease.

Figure 3 shows the effects of *B. monieri* on inflammatory processes.

### 4.3. BM Oxidative Stress, Mithocondrial Dysfunction, and Apoptosis

A common characteristic of neurodegenerative diseases is oxidative stress (OS) resulting from oxidation-reduction reactions in cells, and the human body is exposed to various types of free radicals. When the reactive oxygen species (ROS) are produced in excess, they can generate the oxidative deterioration of molecules involved in advancing aging and neurodegenerative diseases. The neurotoxicity caused by ROS causes changes in neurons, such as the permeabilization of the cell membrane and the reduction of neuronal excitability [143,144]. The generation of free radicals is a fundamental and beneficial process for adequately functioning, protecting, and surviving cells within physiological limits. However, an imbalance in the generation and neutralization of ROS can accumulate harmful intermediate products and cause OS, leading to brain damage [35,145,146,147,148,149,150].

ROS and reactive nitrogen species (RNS), including lipid peroxides, are generated in the brain after brain damage. At the same time, there is a reduction in the levels of antioxidant enzymes such as SOD, catalase, GPx, and glutathione S-transferase (GST), and antioxidant compounds such as glutathione. This imbalance directly contributes to the development of brain injury. BM administration significantly reduced neurological deficits, cerebral infarct edema, and brain volume. Furthermore, it increased brain ATP content, total adenine nucleotides, energy load, and Na+ K+ ATPase activity. It also improves the brain content of GPx, malondialdehyde (MDA), catalase, SOD and reduces the activity of the LOX enzyme, which, in turn, decreases lipid peroxidation [151,152,153,154].

The effects of BM ethanolic extract (BME) on mitigating oxidative stress caused by 3-nitropropionic acid (3-NPA) in dopaminergic cells (N27) and the brains of prepubertal mice were examined. Pretreatment of N27 cells with BME resulted in a significant reduction in the cytotoxicity caused by 3-NPA. Furthermore, BME prophylaxis notably restored the activities of antioxidant enzymes (such as catalase, SOD, GPX, glutathione reductase, and thioredoxin reductase), as well as Na(+), K(+)-ATPase and cycle enzymes of citric acid in the striatum of mice affected by 3-NPA. This suggests that the neuroprotective effects of BME may be related, in whole or in part, to its ability to neutralize free radicals [155].

The impact of BM on the involvement of oxidative dysfunction associated with anxiety state, motor function, and biochemical changes in brain regions of mice chronically exposed to the ecologically relevant herbicide paraquat was investigated. The results revealed that BM supplementation significantly reversed the negative effects induced by paraquat, including exploratory behavior, gait abnormalities, motor impairment, changes in dopamine levels, and cholinergic activity in relevant brain areas. These findings suggest that BM supplementation has a beneficial effect on mitigating behavioral deficits caused by paraquat and cerebral oxidative stress in mice [156].

BM extract significantly attenuates depressive behaviors by normalizing corticotrophic hormone (ACTH) and corticosterone levels in rats. There was also an upregulation of the expression of Brain-derived neurotrophic factor (BDNF), Doublecortin neuronal marker (DCX), and BrdU/NeuN in rats subjected to unpredictable chronic stress and treated with BME. Furthermore, the extract promoted a notable increase in the activity of neuroprotective antioxidant enzymes, possibly through promoting neurogenesis in the hippocampus with increased BDNF levels and offering antioxidant protection against oxidative stress [157].

Shinomol [158] evaluated the potential impact of incorporating BM leaf powder into the diet on endogenous indicators of oxidative stress, redox balance, response of antioxidant defenses (enzymatic responses), protein oxidation, and cholinergic performance in various brain regions of mice in prepubertal stage. Over four weeks, prepubertal stage mice submitted to a diet enriched with BM (0.5% and 1%) showed a significant reduction in basal oxidative indicators (such as MDA levels, production of reactive species, hydroperoxides, and protein carbonyls) in both the cytoplasm and mitochondria of all brain regions analyzed. This effect was accompanied by a reduction in glutathione and thiol levels and an increase in the activities of antioxidant enzymes (catalase, GPX, and SOD). Furthermore, a notable decrease in the activity of the acetylcholinesterase enzyme was observed in all brain areas evaluated, which suggests the potential of BM leaf powder to modulate cholinergic function [158].

Mice with DM2 treated orally with a well-characterized fraction of BM extract (CDRI-08) (used at 50 or 100 mg/kg of body weight) showed a significant increase in spatial memory. This increase correlates with a significant reduction in oxidative stress and the regulation of the α-amino-3-hydroxy-5-methyl-4-isoxazolepropionic acid (AMPA) receptor subunit GluR2 gene expression in the hippocampus. CDRI-08 (150 mg/kg or higher) showed an antidiabetic effect at higher doses and demonstrated the ability to reverse spatial memory impairment. This was accompanied by decreased OS caused by DM2 and the restoration of GluR2 subunit expression levels, bringing them closer to the values observed in standard control and CDRI-08-treated mice. These findings provide evidence on the molecular basis of BM extract’s antidiabetic effect and memory improvement, which may have therapeutic implications [159].

Standardized BM extract can reduce acute OS caused by paraquat and mitochondrial dysfunctions and neurotoxicity in different brain areas of young mice. Oral administration of BM resulted in a significant decrease in the levels of ROS, MDA, and hydroperoxides in several brain areas. Pre-administration of BM also improved OS (restoring ROS, MDA, and peroxide levels) and mitigated mitochondrial dysfunction. The authors suggest that prior use of BM strengthens brain resistance against oxidative stress caused by paraquat and, therefore, could be explored as a preventive measure against oxidation-induced neuronal dysfunctions [35,160].

Other authors investigated the effects of BM ethanolic extract in cells and observed inhibition of Tau aggregation. These cells showed a reduced ROS level and caspase-3 activity. BM extract also promoted the restoration of Nuclear factor erythroid 2-related factor 2 (Nrf2) levels in Neuro2a in formaldehyde-stressed cells. The treatment also decreased GSK-3β phosphorylation in formaldehyde-stressed cells. The authors of this study pointed out that BM extract can play an important role against Tau phosphorylation and aggregation, which can be helpful in the treatment or prevention of Alzheimer’s disease [161].

Finally, BM improved stress tolerance and increased the average lifespan of *Caenorhabditis elegans* during thermal and OS situations. The life expectancy increased by 14–25% compared to untreated control worms [162].

**Figure 4:**
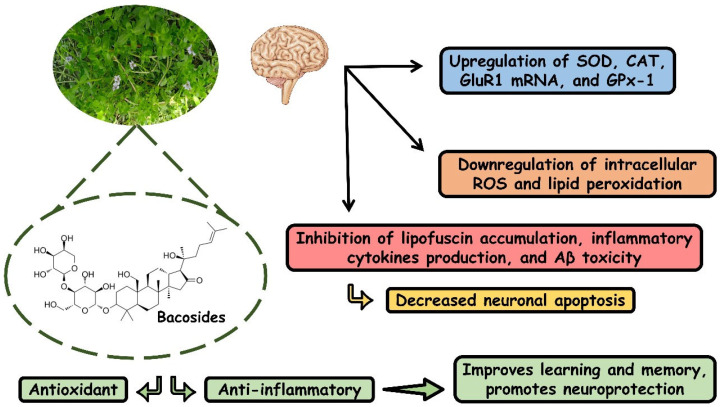
The effects of *B. monieri* on oxidative processes: bacosides can downregulate ROS production and elevate the expression of antioxidant enzymes. CAT, catalase; GPx, Glutathione peroxidase; ROS, Reactive oxygen Species; SOD, superoxide dismutase.

Figure 4 shows the effects of *B. monieri* on oxidative processes.

Mitochondria perform crucial functions for the cell beyond merely synthesizing ATP. In addition to energy generation, this highly dynamic organelle plays vital roles in regulating apoptosis, ferroptosis, and inflammasome activation. The main characteristic of mitochondrial dysfunction is a reduction in the respiratory capacity of mitochondria, accompanied by a decrease in mitochondrial membrane potential under steady-state conditions. Through the execution of these diverse functions, they play essential roles in the development and progression of neurodegenerative diseases such as Alzheimer’s disease and Parkinson’s disease [163,164]. It is known that mitochondria represent a source of ROS. The occurrence of mitochondrial damage or leakage and the accumulation of ROS in cultured neurons can be prevented by treatment with hexane extract of BM, which demonstrates that intact and functional mitochondria are indispensable for adequate neuronal functioning [165].

The effects of BM were also evaluated on mitochondrial dysfunctions in *Drosophila melanogaster*. The authors demonstrated that there was a significant reduction in the activities of the enzymes succinate dehydrogenase (about 23%), complex I–III (about 26%), and complex II–III (about 30%). Interestingly, prophylaxis with BM extract prevented the induction of oxidative stress (OS) by paraquat and restored the activity of ETC complexes, clearly suggesting its specific effect on mitochondria. Although the precise mechanism of action of BM needs further investigation, it may be related to its ability to enhance antioxidant defenses and thus mitigate paraquat-induced OS [166].

Previous application of BM alcoholic extract may offer neural protection against the effects of OS caused by 3-nitropropionic acid (3-NPA) in vitro and in vivo. BM was able to neutralize free radicals in brain mitochondria in vitro completely. Based on these results, it is possible to suggest that the extract could act as a valuable adjunct in brain preservation against neurodegenerative disorders associated with oxidation, notably in scenarios of mitochondrial dysfunction [155].

BM was also administered to diabetic rats once a day for fifteen days at 50, 125, and 250 mg/kg and showed significant reversal of OS and peroxidative damage in the brain and kidneys. These results indicate that BM extract modulates antioxidant activity and increases defense against damage generated by ROS in the brain, mainly in mitochondria and in the kidneys of diabetic rats [167].

In another study, the impact of BM extract on the Nrf2 pathway was examined in rats with memory deficiency induced by okadaic acid, a potent protein phosphatase inhibitor. Okadaic acid targets membrane phospholipids, leading to loss of memory potential, destruction of mitochondrial membranes, and release of factors inducing apoptosis. The doses of BM (40 and 80 mg/kg) were administered one hour before the acid injection and continued daily until the thirteenth day of the experiment. The use of okadaic acid resulted in a significant memory deficit, accompanied by oxidative stress, neuroinflammation, and neuronal loss, associated with reduced expression of Nrf2, heme oxygenase-1 (HO-1), and glutamate cysteine ligase catalytic subunit (GCLC). BM significantly improved memory dysfunction in okadaic acid -treated rats. Furthermore, treatments restored the expressions of Nrf2, HO1, and GCLC, reducing OS, neuroinflammation, and neuronal loss [168].

In a Parkinson’s disease zebrafish model (induced by the compound 1-methyl4-phenyl-1,2,3,6 tetrahydropyridine—MPTP), BM phytochemicals encapsulated in nanoparticles were investigated. Prior administration of nanoparticles significantly reversed the adverse effects of MPTP, resulting in increased dopamine levels and its metabolites and glutathione levels. Furthermore, the activities of GPx, catalase, SOD, and complex I were raised, while MDA levels decreased. There was also an improvement in locomotor activity [169].

Apoptosis, a well-known form of programmed cell death, acts as an essential physiological mechanism restricting uncontrolled cell population growth to maintain tissue stability or eliminate potentially harmful cells, such as those with DNA damage [170]. However, inappropriate positioning of this balancing function may contribute to normal cell growth/proliferation or autoimmune disorders and promote [171,172,173,174].

It was observed that BM restores proteins involved in neural pathways related to cell cycle regulation, programmed cell death (apoptosis), and the functioning of mitochondria [142]. The protective effect of mitophagy mediates the anti-senescence and antiapoptotic activities of BM; if mitophagy is inhibited, there will be no protection of astrocytes in mitochondrial apoptosis induced by ROS and aging of cells treated with BM. Furthermore, BM shows a significant reduction in mitochondria-dependent apoptosis induced by glutamate in the brain. In summary, BM’s anti-aging and anti-apoptosis effects on astrocytes may effectively respond against damage caused by pollution and neurological disorders related to aging [175,176].

The effects of BM extract were assessed concerning the ability to modulate the two N-methyl-D-aspartate receptors (NMDAR) subunits (NR2A and NR2B) and their downstream mediators in the cerebellum of rats with chronic liver failure (ICF) induced by thioacetamide (TAA), and in rats in the TAA group treated orally with 200 mg/kg of BME. NR2A is known to confer neuroprotection, and NR2B induces neuronal death during NMDAR activation. The neuronal nitric oxide synthase-(nNOS-) apoptosis pathway is known to mediate NMDAR-led excitotoxicity. The results demonstrated that the level of NR2A decreased considerably, accompanied by a corresponding increase in NR2B expression in the cerebellum of ICF rats. Additionally, administration of BM extract reversed the ratio between NR2A and NR2B, also restoring the levels of nNOS apoptotic factors in the cerebellum of these rats. These findings suggest that BM extract regulates the expression of NR2A and NR2B subunits, possibly preventing neurochemical changes associated with liver failure [177].

Other studies have shown that bacopaside II blocks the aquaporin-1 (AQP1) water channel, thus impairing the migration of cells that express this protein. AQP1 expression was significantly bigger in HT-29 than in SW480, SW620, and HCT116. Bacopaside II significantly reduced growth by ≥20 µM for HT-29 and ≥15 µM for SW480, SW620, and HCT116. Inhibition of HT-29 at 20 µM was mediated mainly by G0/G1 cell cycle arrest and at 30 µM by G2/M arrest and apoptosis. Inhibition of SW480, SW620, and HCT116 at ≥15 µM was mediated by G2/M arrest and apoptosis. These pioneering results highlight that bacopaside II can inhibit the growth of colon cancer cells, acting through cell cycle arrest and induction of apoptosis [178].

The role of BM against paraquat-induced toxicity was also investigated in the *Drosophila* Parkinson’s Disease model. The results showed that BM attenuates the acute toxicity triggered by venom. BM effects are associated with redox balance, mitochondrial functionality, and reduced apoptosis levels. The mechanisms underlying these effects were identified as the optimization of the active c-Jun N-terminal kinases (JNK) pathway and the reduction of cleaved caspase-3 activity, in addition to the stabilization of the transcriptional expression of genes related to oxidative stress and apoptosis (JNK, caspase-3, damage-associated molecular patterns (DAMP), and Nrf-2). These results highlight the therapeutic efficacy of BM in mitigating paraquat-induced toxicity in the brain, pointing to its potential applications in treating these conditions [179].

**Figure 5:**
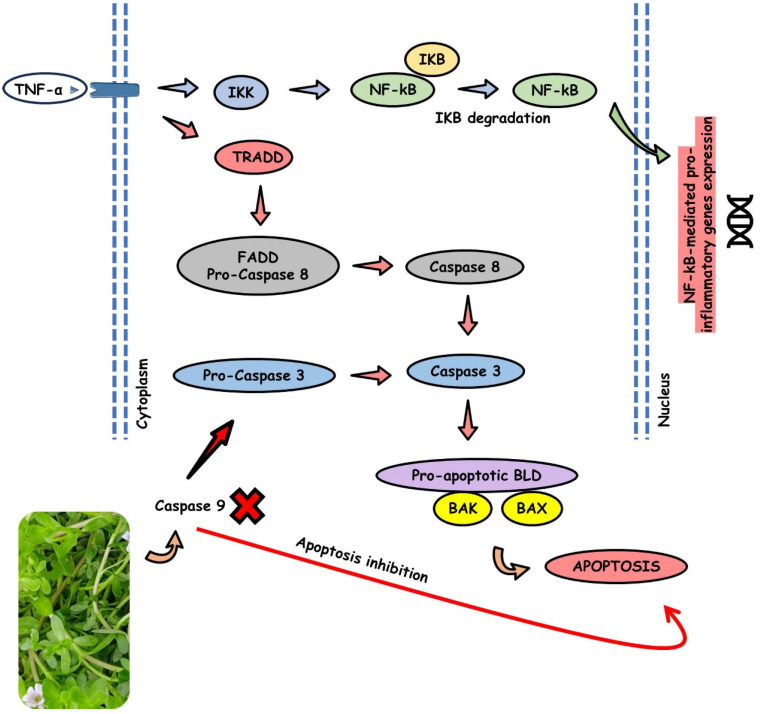
The effects *B. monieri* on the regulation of apoptosis events. *B. monieri* exerts a protective effect on neuronal cells by shielding them from apoptosis, a programmed cell death process, and senescence, the aging-related deterioration of cellular function. This botanical extract demonstrates neuroprotective properties, safeguarding the integrity and viability of neurons. BAX, Bcl-2-associated X protein; FADD, FAS-associated death domain protein; IKK, The inhibitor of nuclear factor-κB (IκB) kinase; NF-kB, Nuclear Factor; TNF-α, Tumor Necrosis Factor-α; TRADD, TNFR1-associated death domain protein.

Figure 5 shows the effects of *B. monieri* on apoptosis events.

The use of BM ethanolic extract in a mice model of Parkinson’s disease induced a remarkable restoration of motor behavior in addition to a significant increase in the levels of catalase, SOD, Glutathione reductase, and GPX. There was also an increase in dopamine levels. Tyrosine hydroxylase immunoreactivity in the *substantia nigra* was substantially reduced in the group treated with MPTP, but this decrease was considerably reversed with the use of BM extract. Furthermore, BM promoted neuroprotection by creating an antiapoptotic environment, indicated by reduced apoptotic activity (Bax and caspase-3) and increased antiapoptotic protein (Bcl2) expression levels. Taken together, the results of this study suggest that BM treatment offers protection to dopaminergic cells in the nigrostriatal pathway against MPTP-induced parkinsonism. This effect was achieved by the modulation of OS and apoptotic mechanisms, which are likely associated with the observed behavioral effects [180].

### 4.4. BM and Neurodegenerative Diseases: Evidence from In Vitro and In Vivo Studies

Neurodegenerative diseases are a diverse set of neurological disorders that have a negative impact on the lives of millions of people. These diseases cause deterioration in the structure and function of neural networks and the loss of neurons. Due to their terminally differentiated nature, neurons cannot renew themselves efficiently. This disrupts central communication circuits, resulting in impairments in memory, cognition, behavior, sensory and/or motor function [181,182,183]. With increasing age on a global scale, the number of neurodegenerative diseases is constantly growing, with Alzheimer’s disease and Parkinson’s disease being the most prevalent [184,185,186,187,188].

Alzheimer’s disease can be considered as the most prevalent type of dementia and is characterized by being a progressive and slowly evolving neurodegenerative disease. The main neuropathological markers are neuritic plaques and neurofibrillary tangles, resulting from the accumulation of beta-amyloid peptide in the most affected areas of the brain [116,183,189,190,191]. Parkinson’s disease is seen as a chronic and progressive neurodegenerative scenario that occurs due to the gradual degeneration of dopamine-producing neurons in the substantia *nigra parspacta* region. This condition is characterized by motor symptoms, including tremors, bradykinesia, akinesia, and postural instability [192,193,194,195].

Medicinal plants play a beneficial role in these cases due to their ability to act through diverse cellular and molecular mechanisms. They can provide relief from inflammatory responses, suppress the functioning of pro-inflammatory cytokines, and improve antioxidant properties [196]. BM amplifies free radical scavenging mechanisms and preserves cells in the hippocampus, prefrontal cortex, and striatum, protecting them against cytotoxicity. Furthermore, it decreases lipoxygenase activity, decreases lipid peroxidation, increases GPX activity, and promotes iron chelation [141,161,197,198].

The dammarane-type triterpenoid saponin and the aglycone unit in triterpenoid saponins (jujubogenin or pseudojujubogenin) (bacosides) can repair damaged neurons, stimulate kinase activity, restore synaptic function, and improve nerve transmission [95,199].

The dysregulation of acetylcholinesterase activity may contribute significantly to the development of neurodegenerative disorders. Chemical structures of BM were retrieved from PubChem database and subjected to in silico and in vitro approaches to analyze its modulating properties in acetylcholinesterase function and its possible use in neurodegenerative disorders treatments. The results showed that BM phyto- compounds such as quercetin, wogonin, apigenin, and bacopaside X can dose-dependent inhibit acetylcholinesterase, and the most efficient of them is the bacopaside X. Therefore, BM presented neuroprotective activity and may complement neurodegenerative disorders treatments [36].

It was investigated if the supplementation of BM extract to mothers exposed to social stress during gestation can modify the effect of the stress on their children’s neurobehaviour, antioxidant defense gene expression, telomere length, and telomere biology. During the tests, timed pregnant rats were subjected to social stress in gestational day 16–18, and then a group of stressed pregnant rats received BM extract every day from gestational day 10 until their pup attained postnatal day 23. It was observed that the supplementation of BME dampens maternal stress. Thus, its effect on neurobehaviour, antioxidant defense gene expression, and telomere biology is minimized in their offspring is also minimized [200].

With the cholinergic system, memory can be modulated through neuronal cells and also through non-neuronal cells, such as microglia, that can influence synaptic function and plasticity. *Withania somnifera* (L.) Dunal (WS) and BM are traditionally used for the temporary relief of symptoms of stress, which chronically can cause memory deficits. The study evaluated if choline activity can be enhanced by the association with WS and BM extracts (adaptogens). The tests were made on an in vitro model of corticotropin-releasing hormone-induced oxidative stress on microglial BV2 cells, which reduced BV2 cell viability, induced morphological changes, and neurotoxicity, and also increased the production of reactive oxygen species and dysregulated antioxidant protein. The WS and BM extracts, when combined with choline, neutralized the corticotropin-releasing hormone effects, reducing microglia-induced oxidative stress, proving to be a great intervention in cases of memory disturbances caused by chronic stress [201].

A study described the isolation and identification of seven known phenyl glycosides and one new, the bacomoside D3, compounds isolated from BM, traditionally used as a neural tonic and memory enhancer to relieve acute pain and inflammation. The compounds were evaluated for antioxidant and anti-inflammatory activities, and compounds four and five exhibited strong 2,2-difenil-1-picrilhidrazil radical scavenging activity, while compounds two and five significantly inhibited TNF-*α* production in lipopolysaccharide- stimulated RAW264. Also, the compounds could efficiently inhibit oxidants by interfering with the 2,2-diphenyl-1-picrylhydrazyl activity in silico [202].

A neuroprotection experiment analyzing the phytocompounds of bacopaside X and quercetin was developed using six groups of mice with different interventions. Visual behavioral assessment using the Morris water maze showed that mice in the diseased model group (scopolamine) moved slowly toward the platform and exhibited greater thigmotaxis behavior than the treatment and control groups. Also, the concentration of nitric oxide, GSH, MDA, and mRNA levels of different marker genes, including ChAT, IL-1α, IL-1 β, TNF α, tau, and β secretase, improved in treatment groups concerning the diseased group. Therefore, both bacopaside X and quercetin presented antioxidant and neuroprotective properties [203].

Microtubule affinity regulating kinase 4 (MARK4) participates in the development of Alzheimer’s disease by hyperphosphorylating tau protein and is a drug target for Alzheimer’s disease. BM extract is known by its neuroprotective properties and is commonly used as a memory enhancer and brain tonic for the treatment of neurological disorders. In this study, the inhibitory effects and binding affinity towards the MARK4 of Bacopaside II, a major component of BM, was evaluated. The results showed that Bacopaside II has binding affinity for MARK4 and can inhibit kinase activity. The compound also binds strongly to the active site pocket residues of MARK4 and a number of hydrogen bonds remain stable throughout the molecular dynamics simulation trajectory [84].

The use of BM in rats is capable of suppressing the levels of pro-inflammatory cytokines (TNF-α, IL-1 and 6, COX-1 and 2, and iNOS), reducing the levels of α-synuclein and decreasing the production of ROS. In Parkinson’s disease, it was shown that the ethanolic extract of the plant has antiapoptotic properties in dopaminergic neurons. These results indicate that BM can inhibit inflammation in different brain regions, making it a promising source for developing new therapies to treat various CNS disorders [204].

The neuroprotective effects of bacosides were also investigated in age-associated neurodegeneration and their impact on the prevention of Senile Dementia of Alzheimer’s Type (SDAT). The optimal dose of bacosides without adverse effects was selected by screening their dose-dependent activity on the aging biomarker lipofuscin and the SDAT biomarker neurotransmitter acetylcholine in the aged female Wistar rat brain. The results of this study suggested that bacosides have the potential to be a promising therapeutic intervention to mitigate the detrimental effects of aging and prevent age-related conditions, including SDAT [205].

An interesting study compared the effects of BM (10 and 40 mg/kg) and deprenyl (1 and 2.5 mg/kg), a mono-amino oxidase (MAO-B) inhibitor (and a common drug used to treat dementia) in Wistar rats. The results showed that both BM and deprenyl increased catalase activity and the expression of p-tyrosine hydroxylase, nerve growth factor, and NF-kB in the spleen. On the other hand, the activities of SOD, catalase, and GPX in organs such as the thymus, mesenteric lymph nodes, heart, and several brain areas (frontal cortex, medial basal hypothalamus, striatum, and hippocampus) were differentially modulated by BM and deprenyl. It was observed that BM, when administered alone, increased Nitric oxide (NO) production in the spleen. Thus, the results suggest that using these compounds reduces free radical levels and improves the neural-immune functions of the brain, heart, and lymphoid organs. This may be particularly relevant for health promotion, especially in contexts related to neurodegenerative diseases [206].

Another study verified the effects of in vitro incubation of lymphocytes from spleens of different age groups in F344 rats, both with the use of BM and donepezil. It inactivates the cholinesterases, thus inhibiting hydrolysis of acetylcholine), on the following variables: proliferation of T lymphocytes induced by concanavalin, production of cytokines, and antioxidant enzyme activities. It was observed that the age-related decline in T lymphocyte proliferation induced by concanavalin was not reversed by treatment with BM and donepezil. However, donepezil alone further reduced lymphocyte proliferation in young rats. In contrast, lower doses of BM treatment were able to reverse the age-related decrease in concanavalin-induced IL-2 and IFN-γ production by splenocytes. The extracellular signal-regulated kinase 1/2 (p-ERK1/2) and p-CREB expressions in splenocytes increased after BM and donepezil treatment in old and middle-aged rats. Conversely, the age-related decline in p-Akt expression was reversed by BM treatment alone in middle-aged and aged rats. These results suggest that both BM and donepezil have distinct age-related effects on cell-mediated immune responses. This occurs through selective modulation of the activities of antioxidant enzymes and intracellular targets, which may interfere with the therapeutic efficiency of these drugs in neurodegenerative diseases [207].

BM has shown neuroprotective effects in animal and in vitro studies, but studies in human patients with Alzheimer’s Disease have been conflicting. Therefore, a review presented difficulties in designing clinical trials of BM in neurodegenerative diseases was conducted, and their evidence-based recommendations were proposed. It was found that BM trials need improvement, particularly in effect size and sample size estimation, and assessment and outcomes measures need a more comprehensive approach and newer scales for diagnosing and monitoring prodromal AD. Following the CONSORT and STROBE guidelines would enhance the quality of evidence and the evaluation BM’s efficiency in Alzheimer’s disease/prodromal Alzheimer’s disease and other neurodegenerative dementia treatments. The studies should also implement more randomized controlled trials with an appropriate sample size of accurately diagnosed AD/prodromal AD patients, administering a recommended dosage of BM and for a predetermined amount of time estimated to achieve sufficient power for the study. Additionally, more sensitive cognitive scales, especially for prodromal AD, should be developed, and BM evaluated with more rigorous standards [208].

### 4.5. BM and Neurodegenerative Diseases: Results of Clinical Trials

Many studies have shown the effects of BM on brain disorders such as memory impairments, dementia, Alzheimer´s disease, Parkinson´s disease, hyperactivity, depression, learning capacity, and anhedonia (Table 1). In a very recent clinical trial, the authors investigated the effects of BM on healthy subjects for three months. Mood and cognition were evaluated respectively by the geriatric depression scale (GDS) and the Montreal Cognitive Assessment (MoCA). Their results showed that BM can exert antiinflammatory actions due to inhibiting NFκB and improving CREB. The limitations of this study include mainly unblinded and no placebo comparison, the small sample, limited diversity (96% white), and the inclusion of mostly females (75%) [56].

**Figure 6:**
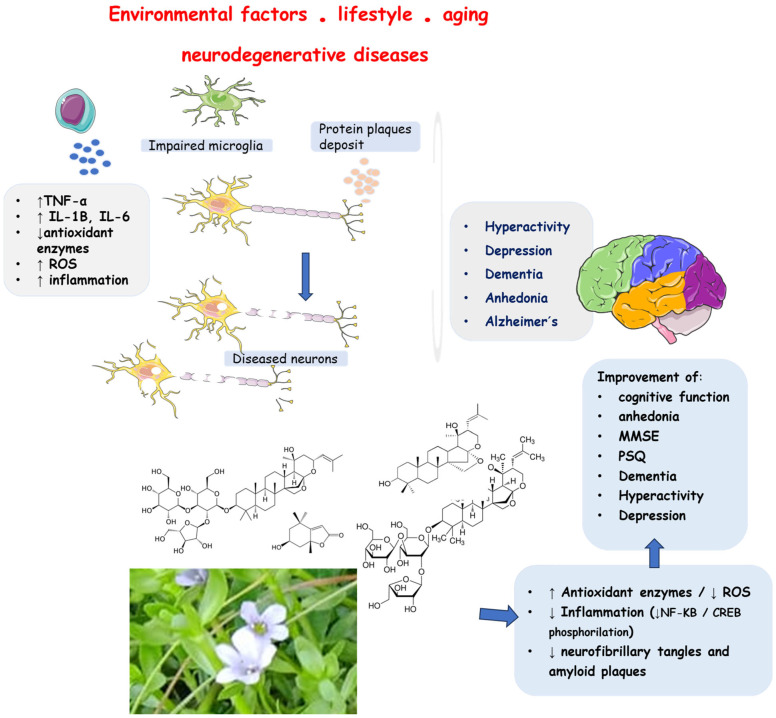
General effects of the use of *B. monieri* on humans. The plant can improve general brain functions as hyperactivity, depression, dementia and Alzheimer´s disease due to several actions such as anti-inflammatory and antioxidant. CREB, Cyclic AMP response element-binding protein; MMSE, Mini-Mental State Examination; NF-KB, Nuclear Factor-KB; PSQ, Perceived Stress Questionnaire; ROS, Reactive oxygen Species. ↑: increase; ↓: decrease.

Figure 6 summarizes the effects of the use of BM on humans.

Santos et al. (2023) [57] analyzed the effects of BM in patients with Parkinson’s disease and showed that the plant could improve emotional functions. Although this is an interesting study, the authors did not mention the gender and age of participants. Moreover, the sample size is small, which can interfere with the study’s results.

Kean et al. (2022) [58] evaluated the effects of BM in male children and showed a cognitive improvement. Although the authors include many limitations for this trial, this was a well-done study, with adequate sample size and adequate follow-up, showing that BM can be considered in the treatment of cognitive disorders and hyperactivity in children. The study protocol regarding the efficiency of BM extract (CDRI 08) was registered by the same authors [209].

Other authors also investigated the effects of BM on healthy patients. The authors related that they found no compounds and metabolites of BM in the plasma and urine samples of treated patients but were detected in the feces sample. Although the study is interesting, the number of participants is small, and the authors did not present the participants’ gender. Furthermore, the study did not show sample calculation or clearly demonstrate demographic data. These biases can interfere with the interpretation of the results [59].

In a trial, the authors showed that the use of BM can improve anhedonia and major depression [60]. These are interesting results; however, the study was not blinded, and a lack of a placebo-controlled study may have compromised the validity of the results.

In another study [61], the effects of MB were compared to donepezil in patients with Alzheimer’s disease (diagnosis confirmation by magnetic resonance imaging of the brain, brain tomography, and evaluation of cerebrospinal fluid biomarkers, such as β amyloid and total tau). This was a very well-designed study with a small sample. However, the authors performed sample calculation. Due to these reasons, it is possible to say that this phase-two randomized clinical trial demonstrated that there were no significant differences between BM and donepezil after twelve months of treatment. The authors suggest that larger and multicentric phase-3 clinical trials are necessary to verify if BM is superior to donepezil in this kind of patient.

In another clinical trial, the effects of a mix containing BM, astaxanthin, lycopene, and cobalamin improved several aspects of cognitive performance in healthy elderly participants. Although the study is well-performed, and the findings are promising in suggesting that regular intake of this mix can effectively reduce cognitive modifications related to brain aging, it is not possible to say that these effects are related solely to BM [62].

In a pilot study, BM was investigated as a memory enhancer in elderly patients. All patients showed a rise in cognitive fitness with no significant adverse effect [63]. This study included a very small sample, and many biases were found (Table 2).

Cicero et al. [64] evaluated the effects of BM in patients with Mini-Mental State between 20 and 27 and self-perceived cognitive decline. The study showed that the active treatment arm’s MMSE and PSQ Index significantly improved. Although this is an interesting study, the authors did not present the gender of the patients, and the sample size was small. These biases can interfere with the interpretation of the results. Moreover, BM was combined with other nutraceuticals; therefore, the observed effects cannot be attributed solely to this plant.

Another clinical trial included students with high cognitive functions and showed that BM could significantly improve cognitive functions along with a significant increase in serum calcium levels The cognitive performance was validated by examining various neuropsychological tests (logical memory test, digit span memory task, etc.). The findings revealed no change in the brain’s attention and sensory-motor performance, suggesting that BM can decrease students’ distractibility but also improve cognitive functions. The authors conclude that memory and learning are complex events involving several neurotransmitters and factors. Due to these reasons, further studies are necessary to establish BM’s role in cognitive functions [65]. In this study, the authors did not mention the percentage for each gender of the included patients, and the sample size was small.

In another interesting study, the authors studied the effects of BM in healthy and SDAT patients. The results demonstrated that there was a significant amelioration in cognitive functions and a decrease in oxidative stress and inflammation in the evaluated participants. Although this is an exciting study, it did not present the gender of patients, and BM was mixed with other components. Therefore, the observed effects cannot be attributed solely to BM [66].

The effects of BM were tested in children with attention-deficit hyperactivity disorder (ADHD), and the outcomes showed that there was a reduction of ADHD symptoms, attention deficit, learning problems, impulsivity, and psychiatric problems. Although this is also an exciting study, the sample size was small [67].

Another study showed positive cognitive and mood effects of BM in healthy people. Furthermore, the use of the plant reduced the levels of cortisol. One relevant bias for this study was the small sample size [68].

In healthy patients, the effectiveness of BM extract (KeenMind^®^) on cognitive performance was investigated. The authors investigated a series of well-validated neuropsychological assessments in this double-blind, placebo-controlled trial. The results were promising regarding the use of BM in early information processing, verbal learning, and memory strengthening compared to the use of a placebo. However, the sample size of this study was also small, reducing the power of evidence for the results [69].

The effects of BM was also performed in attention, cognitive processing, working memory, and cholinergic and monoaminergic actions in healthy participants [70]. The results are promising, showing that BM can improve cognitive functions.

Additionally, Morgan et al. (2010) examined the influence of BM on memory performance in older individuals and demonstrated enhancements in memory among healthy patients. This study featured a sufficient sample size and comprehensive follow-up investigation [71].

Some researchers showed that the use of BM in patients with no clinical signs of dementia is related to a reduction in depression and anxiety symptoms. Moreover, BM can also reduce heart rate. This trial also presented an adequate sample size and a follow-up investigation [72].

The actions of an enriched phytochemical mix of BM extract (BACOMIN^®^) were evaluated in subjects with self-reported memory impairment (MMSE score >24) for at least one year. This study is interesting since the authors analyzed neuropsychological assessment (memory, attention, and information processing speed) at baseline, after twelve, and twenty four weeks; however, the results can be biased since the loss of participants was relevant [73].

Regarding neuropsychological tests, a study investigated the effects of BM (KeenMind^®^) or placebo. This study included an adequate sample size. However, a possible bias is that the neuropsychological variables were performed according to computerized processing speed tasks, which can be contrasted to pencil and paper tests employed in other studies [74].

Other authors investigated the effects of BM in patients with age-associated memory impairment (no presence of dementia or psychiatric illness). Although the results are promising, the small number of patients included can have decreased the statistical power and the interpretation of the results [75].

The use of BM in healthy patients was associated with a significant improvement in the retention of attention verbal and visual short-term memory. This is also an interesting study with promising results. The sample size and follow-up are adequate to extrapolate the conclusions [76].

KeeMind^®^ was also investigated for three months, ameliorating the accuracy in more complex cognitive tasks [77]. Neuropsychological evaluations were performed at baseline, five and twelve weeks after KeeMind^®^ administration. The results revealed significant improvement in learning rate, the speed in the processing of visual information, and memory consolidation.

In another review, the authors intended to evaluate if BM had clinical efficacy and safety in patients exhibiting mild cognitive impairment or mild, moderate, or severe dementia due to Alzheimer’s disease. Only five trials included in this review studied the effects of the plant alone or in association with other medications, such as donepezil. The doses of the extract varied from 125 to 500 mg twice a day. The evaluated outcomes were Mini-Mental State Examination scores, Cognitive subscale scores of the Alzheimer’s disease assessment scale, and cognitive tests. All of the included trials were judged as showing high bias risk. The evidence was found to be of very low certainty. Analysis of data from five trials suggested that there was no notable variance in efficacy between BM and either a placebo or donepezil for the treatment of Alzheimer’s disease or mild cognitive impairment. Furthermore, no significant safety issues were identified in the trials encompassed within this review [82].

### 4.6. Constituents of BM and Neurodegenerative Diseases: Results of Clinical Trials

The overall effects of BM in neurocognitive diseases are probably related to the presence of bacoside A, bacoside B, and bacosaponins. As already mentioned, these bio compounds can decrease inflammation process, OS, mitochondrial dysfunction, neuronal oxidative stress, inhibition of α-synuclein aggregation, and dopaminergic neuronal degeneration, and can substantially improve cognition and learning behavior. Due to these actions, BM can promote neuroprotective effects against Parkinson´s disease since it ameliorates oxidative stress and neuroinflammation and can augment dopamine levels [192]. Bacoside A, bacoside B, bacopasaponin C, bacopaside I and II, asiatic acid, betulinic acid, loliolide, and quercetin can modulate intracellular signaling pathways, neuronal/glial plasticity, neurotransmission, neurogenesis, cerebral blood flow, energetic metabolism, reduction of oxidative stress, endoplasmic reticulum stress, inflammation, and apoptosis. These effects can benefit brain impairment and, therefore, can reduce depression, cognition, memory, epilepsy events, dementia, Alzheimer’s disease, and Parkinson’s disease [45].

Banerjee et al. [85] built a review regarding the effects of BM and its bioactive compounds (bacosides) in the management of neurological impairment. The authors critically evaluated the effects of bacosides on Alzheimer´s and Parkinson´s diseases, epilepsy, autism, encephalomyelitis, encephalomyelitis, and memory loss. Bacosides A and B can improve memory and decision-making ability, helping individuals stay calm. In in-vitro and in-vivo studies, bacosides delayed the onset of Alzheimer´s disease and could also control several other pathophysiological activities of this disease. Regarding neurodegeneration, in vitro models of ischemia were treated with bacosides, and in vivo (mice) treated with BM (25 μM of bacopaside I) promoted neuroprotective effects against cell damage due to oxygen and glucose-deprivation (OGD)-induced nerve. Bacosides could also prevent apoptosis. In rats, BM prevented dopaminergic neurons due to the decrease of antioxidant enzymes such as superoxide dismutase and catalase. Moreover, it decreased the accumulation of alpha-synuclein. Bacoside A alters these changes, hence proving its medicinal significance against epilepsy. During epilepsy, a reduction in metabotropic glutamate-8 receptor is observed, but the administration of BM increases it. In a mice model of Parkinson´s disease, BM significantly increased hydroxylase activity, caspase 3, the expression of neurogenic genes (in the *substantia nigra*) and reduced oxidative stress.

## 5. Safety

The studies found in the literature show that the use of BM is unrelated to toxicity. Some authors have shown that administering a single acute dose orally at 5000 mg/kg did not show toxicity in the animals monitored for fourteen days. In the chronic toxicity evaluation, male and female rats received oral doses of 30, 60, 300, or 1500 mg/kg daily for 270 days. The behavior and overall health of the rats were carefully examined. After the study period, the results showed no differences regarding controls. All of the results for other parameters that were assessed remained within the normal range: no mortality, no changes of skin, fur, eyes, mucous respiration, membranes, autonomic or central nervous systems circulatory system, were observed after the acute dose; body weight significantly increased in the treated group, and no significant modifications on histopathological of the internal organs, were seen. On the other hand, in the chronic test, the weight of some organs decreased in the groups that received BM [210].

In another experimental study with rats, the use of alcoholic extract of BM produced no adverse effects at 500 mg/kg [211].

Although the use of BM extracts has been considered safe, it is worth mentioning that the use by humans can be related to some adverse events (side effects) such as diarrhea, nausea, increased stool frequency, abdominal cramps, stomachache, stomach flu, asthenia, arthralgia, headache, dizziness, anxiety, insomnia, dry mouth, increased appetite; headache, palpitations, and worsening cystitis, arthritis, and tendon surgery. On the other hand, no modifications in laboratory parameters were found along with the intervention studies [56,61,71,77].

According to Aguiar and Borowski, the standard experimental doses in humans are between 150 and 3000 mg daily. These authors report that the most common clinical adverse events are mild gastrointestinal complaints, but long-term randomized clinical trials are still necessary to achieve secure doses and reduce adverse events [212].

In a review studying the safety of BM and other plants, the authors point out that using plants or supplements for cognitive performance has attracted much attention from the community. Regarding BM, the authors included some of the clinical trials reviewed in our study and showed that gastrointestinal symptoms are the main adverse events related to using this plant extract. However, they also pointed out the need for more well-performed and larger sample clinical trials with BM in humans [213].

## 6. Conclusions, Strengths, Limitations, and Future Directions

This systematic review provides a comprehensive overview of the existing evidence on the beneficial effects of BM on brain health and neurodegeneration. Although the existing clinical trials show that the use of BM is promising in many brain disorders, there is still an urgent need for new studies that can define better doses, adequate pharmaceutical formulations, the definition of standard extract formulation, possible adverse events, or drug interactions. These studies should be performed in larger populations, appropriate age and gender groups, and well-defined diseases. Therefore, it would be possible to establish systematic clinical and safe uses.

The strength of this systematic review is that it shows, for the first time, all of the studies that evaluated the effects of BM in diseases related to neurodegenerative conditions. Moreover, an extensive literature search was performed, and several articles were included, obeying PICOS format. In this sense, we brought to light the overall and current scenario of the effects of BM in humans. On the other hand, there are limitations that should be considered. First of all, in general, the included studies presented a small sample size, and some did not use a blinded investigation or comparison with a placebo. Furthermore, a wide range of doses were used (from 150 to 640 mg), and the duration of the treatment ranged from four weeks to six months. The lack of high-quality investigation in several trials was highlighted, suggesting the need for further well-conducted and adequate small-sample studies in the future.

However, several gaps and limitations must be addressed in future research. First, the quality and rigor of the clinical trials included in the review vary widely, and some have small sample sizes, short durations, or lack adequate control groups. Therefore, more well-designed, randomized, placebo-controlled, double-blind trials are required to confirm the efficacy and safety of BM in different neurodegenerative conditions and populations. Secondly, the optimal dose, frequency, and duration of BM administration have not been clearly established, and the bioavailability and pharmacokinetics of BM and its active compounds are not well understood. Thus, more studies are needed to determine the optimal parameters for BM intake and to explore the pharmacological mechanisms of BM and its metabolites in the brain. Third, the molecular targets and pathways of BM in modulating neuroinflammation, oxidative stress, apoptosis, neurogenesis, and neurotrophism have not been fully elucidated, and the interactions between BM and other genetic, epigenetic, environmental, and lifestyle factors have not been investigated. Moreover, neurodegenerative diseases are linked to neuroinflammation and viral infections, with studies suggesting that exposure to common viral pathogens increases the risk of conditions such as Alzheimer’s disease and Parkinson’s disease [214,215,216,217]. Therefore, more mechanistic studies are needed to unravel the complex network of BM and its effects on the brain, and to identify potential biomarkers and predictors of BM response. Fourth, the long-term effects and outcomes of BM on brain health and neurodegeneration have not been assessed, and potential adverse effects or interactions of BM with other drugs or supplements have not been reported. Thus, more longitudinal and follow-up studies are needed to evaluate the durability and safety of BM in the prevention and treatment of neurodegenerative diseases. By addressing these gaps and limitations, future research can advance the knowledge and application of BM as a promising natural product for brain health and neurodegeneration.

## Figures and Tables

**Table 1:** Studies showing the effects of *B. monnieri* or its derivatives on human health.

Model/Country	Population	Intervention/Comparison	Outcomes	Side Effects	Ref.
Open-labeled study. USA	35 healthy subjects (75% female), 60–78 years, with an education corrected score of 25 or above on the MoCA and a score of 9 or below on GDS.	Participants receive BM (CDRI 08) 320 mg/2x/day/3 months.	After 3 months, GDS and MoCA did not change significantly; the delayed recall subscale showed a significant improvement; mBDNF and proBDNF levels did not exhibit significant changes. There was a significant increase in CREB phosphorylation and a significant reduction of NF-κB phosphorylation.	Nausea, stomach pain, and diarrhea.	[56]
The study was a primary, interventional, controlled, parallel, double-blind clinical study. Brazil	20 volunteers with Parkinson’s disease, 13 men and 7 women, 69–85 years	Patients received BME (225 or 450 mg/day) or placebo for 90 days. Motor activity was evaluated before treatment and 30, 60, and 90 days after, and PDQL questionnaire was applied.	The delta percent (Δ%) showed that there were time-dependent improvements in emotional function.	NR	[57]
The study was a 14-week randomized, placebo-controlled, double-blind, clinical trial (plus a placebo run-in and run-out phases). Australia	112 boys, 6–14 years, exhibiting inattention and hyperactivity against placebo, with 93 datasets available for analysis	Patients received BM (CDRI 08^®^) for 14 weeks: 160 mg of BM or placebo (body weight 20–35 kg) or 320 mg/day (if over 35 kg)/14 weeks.	No significant differences in behavior between groups was observed. BM reduced error-making in children and augmented the speed of reaction time. There was significant amelioration in cognitive flexibility, executive functioning, interpersonal problems, and sleep routine.	NR	[58]
Double-blind, randomized, placebo-controlled study. Thailand	48 healthy patients, 55–80 years, and with Thai ethnicity, read or write in Thai.	Subjects received one bottle of placebo/day/2 weeks (placebo run-in period). Participants were then divided into the treatment (1 bottle of BM essence/day/12 weeks) and placebo group.	BM essence improved memory speed (assessment by computerized tests). BM active compounds and metabolites were not detected in the plasma and urine samples but were detected in the feces sample.	NR	[59]
Randomized study Italy	42 patients (64% women; >47 years) with a significant degree of anhedonia (SHAPS score ≥ 3) and with major depression (DSM 5.0) with no satisfactory response after 4 weeks treatment with citalopram.	Patients received citalopram or citalopram associated with BM (300 mg)/4 weeks.	There was a significant improvement in the Hamilton depression rating scale, SHAPS, strength, and difficulties questionnaire.	NR	[60]
Randomized Double-Blind Parallel Phase 2b Study. India	48 AD and MCI-AD patients, including cognitive and quality of life outcomes.	Patients received either BM (300 mg) or donepezil (10 mg) for 1 year.	No difference in the rate of change in memory scale was observed at 3, 6, and 9 months. In the last follow-up, significant difference in the change in PGIMS score between BM and donepezil.	No significant difference in AD in both groups: nausea, diarrhea, asthenia, arthralgia, headache, dizziness, anxiety, and insomnia.	[61]
Randomized, double-blind, controlled, parallel arm study. Italy	80 healthy subjects, 60 years or more; 25 men and 55 women).	After a 1-week run-in period, subjects received a mix of antioxidants (BM, astaxanthin lycopene, and vitamin B12) or placebo daily/8 weeks.	The consumption of the mix can be effective in counteracting cognitive discount related to brain aging, such as improvement in trial-making test (TMT) scores, and augmentation in letter fluency evaluated by the verbal fluency test.	Exacerbation of sinusitis	[62]
Pilot study.	12 participants with dementia > 18 years	Participants with dementia (all grades) received 250 mg of BM/3 months	All patients showed a positive response: GDS was: 4.42 before BM treatment and 1.92 after 3 months.	NR	[63]
Double bind, cross-over trial with a placebo comparison Setting: outpatient clinical practice. Italy	30 elderly subjects, 61–69 years, with basal MMES 20–27 and self-perceived cognitive decline.	Participants received a mix of nutraceuticals based on BM (320 mg), L-theanine 100 mg, *Crocus sativus* 30 mg, biotine 450 mcg, vitamin B6 (9.5 mg), folic acid 400 mcg, vitamin B12 (33 mcg), vitamin D (25 mcg) and cupper (2 mg).	There was a significant improvement in the SRDS scores, in both groups. MMSE and PSQ Index significantly improved in the active treatment arm.	NR	[64]
Randomized Placebo-Controlled Trial non-crossover, parallel trial conducted on outdoor basis. India	60 health medical students, 19–22 years.	Participants received BM (Bacognize^®^) (150 mg) or placebo 2x/day/6 weeks	The use of BM significantly ameliorated cognitive function, and a significant rise in serum calcium was also observed.	NR	[65]
Randomized, double-blind placebo and active-controlled clinical trial. India	109 healthy subjects, and 123 SDAT patients being over 60 years	Participants were divided into Group A: healthy elderly subjects who received placebo; Group B: healthy elderly subjects who received the test formulation (*BM, Hippophae rhamnoides*, and *Dioscorea bulbifera;* total dose of 500 mg); Group C: SDAT patients treated with donepezil; and Group D: SDAT patients treated with the test formulation. BM (500 mg); donepezil 10 mg, 2x/day/12 months	The administration of BM resulted in significant improvement of cognitive functions and reduction in oxidative stress and inflammation related to neurodegeneration in healthy elderly subjects, comparison to placebo, and in SDAT patients, comparison to donepezil.	NR	[66]
Clinical trial conducted as an open-label study. India	31 children, 3 women and 28 men, 6–12 years, with an age of onset of ADHD before 7 years, as defined by the DSM-IV criteria for ADHD.	Patients received BM extract (BacoMind^®^) 225 mg/day/6 months.	There was a significant reduction in scores of ADHD symptoms (except for social problems), attention-deficit, learning problems, impulsivity, and psychiatric problems.	NR	[67]
Double-blind, placebo-controlled cross-over study. Australia	17 healthy patients, 13 women and 4 men, 18–44 years.	Patients received 320 mg of BM, and 640 mg of BM 1 h, and 2 h after consuming a placebo. A 7-day washout separated the treatments.	There were positive cognitive effects and mood effects; cortisol levels were reduced.	NR	[68]
Double-blind, placebo-controlled. Australia	24 healthy patients, 20 women and 4 men, aged 18–56 years, who completed a cognitively demanding series of tests.	Patients received BME (KeenMind^®^-CDRI 08), 320 mg or 640 mg in a cross-over design.	There was no effect in attenuating task-induced ratings of stress and fatigue or cardiovascular activity.	NR	[69]
Randomized double-blind placebo-controlled design. Thailand	60 healthy adults; mean age 62.62 years, 23 men and 37 women.	Participants received BM (300 and 600 mg) or placebo/1xday/12 weeks	BM improved working memory and there was improvement in cognitive processing, attention, and working memory.	NR	[70]
Randomized, double-blind, placebo-controlled trial. Australia	98 healthy volunteers, 52 women and 46 men, over 55 years.	Patients received BM (BacoMin), 300 mg/day, or an identical placebo for 12 weeks.	There was an improvement in memory retention and acquisition.	Nausea, increased stool frequency, and abdominal cramps	[71]
Randomized, double-blind, placebo-controlled trial (with a placebo run-in of 6 weeks). Australia	54 patients, 60% women and 30% men, without clinical signs of dementia, 65 years or older.	Patients received BM extract, 300 mg/day or a similar placebo for 12 weeks.	There was an amelioration of AVLT delayed word recall memory scores compared to placebo. Depression, anxiety, and heart rate decreased.	Stomach upset	[72]
Randomized double-blind placebo controlled. India.	65 individuals, 50–75 years with self-reported memory impairment (MMSE > 24) for at least 1 year. Neuropsychological evaluation was performed at 0, 12, and 24 weeks. Outcomes were analyzed for attention, memory, and speed of information processing.	Patients received BacoMind^®^ 450 mg/day/12 weeks or placebo and more 12 weeks of placebo (24 weeks study).	BM ameliorated performance in the investigated tests associated with attention and verbal memory. Significant interaction was observed between group and time in the digit span backward evaluation (*p* = 0.008), paired associates dissimilar delayed recall investigation (*p* = 0.047), list learning delayed recall test (*p* = 0.014), and in the visual retention-I test (*p* = 0.035).	NR	[73]
Double-blind placebo-controlled randomized trial. Australia (?)	107 healthy adults (62 completed the study; 21 males and 41 females), 18–60 years	Participants received 2 × 150 mg of BM (KeenMind^®^) or placebo. Neuropsychological testing was performed at 0 and after 90 days.	BM significantly ameliorated performance on the ‘working memory’ factor (specifically spatial working memory accuracy).	NR	[74]
Double-blind, placebo-controlled randomized study. India	40 patients > 55 years with complaint of memory loss in daily activities (Wechsler Memory Scale: logical subset score < 6)	Patients received 125 mg BM extract or a placebo/2x/day/12 weeks, followed by the placebo for both groups for another 4 w. Outcomes: MMSE and Wechsler Memory Scale (visual reproduction, logical memory, and learning).	BM led to significant amelioration of logical memory, mental control, and paired associated learning.	NR	[75]
Double-blind randomized, placebo control study. Australia	76 healthy adults, 48 women and 28 men, 40–65 years.	First session: 3 months supply of capsules 450 mg for patients > 90 kg and 300 mg for <90 kg). Second session: 3 months later patients were instructed to take not the capsules. Third session was 6 weeks after the end of trial session.	There was a significant effect on the retention of new information. Rate of learning attention, verbal, visual short-term memory retrieval of pre-experimental knowledge, everyday memory function and anxiety levels were unaffected.	NR	[76]
Double-blind placebo-controlled independent-group design. Australia	46 healthy volunteers, 18–60 years; 11 males and 35 females.	Participants received Keenmind^®^ (standardized: >55% of combined bacosides A and B), 2 × 150 mg or placebo/12 weeks	There was a significant improvement in learning rate, the speed of visual information processing, and memory consolidation.	Dry mouth, increased appetite, headache, palpitations	[77]

Abbreviations: ADHD: Attention-deficit hyperactivity disorder; AVLT: Rey Auditory Verbal Learning Test; BM: *Bacopa monnieri*; BME: *Bacopa monnieri* extract; CREB: Cyclic AMP response element-binding protein; GDS: Global Deterioration Scale; DSM-IV: Diagnostic and Statistical Manual of Mental Disorders; MMSE: Mini-Mental State Examination; MoCA: Montreal Cognitive Assessment; NR: Not reported, PGIMS: postgraduate institute memory scalePSQ: Perceived Stress Questionnaire; SDAT: senile dementia of Alzheimer’s type; SHAPS: SHAPS, Snaith–Hamilton Pleasure Scale.

**Table 2:** Risk of bias assessment included in this review.

Study	Question Focus	Allocation Blinding	Double- Blind	Losses (>20%)	Prognostic or Demographic Characteristics	Outcomes	Intention to Treat	Sample Calculation	Adequate Follow-Up
[56]	Yes	No	No	No	Yes	Yes	No	No	Yes
[57]	Yes	Yes	Yes	No	Yes	Yes	No	No	Yes
[58]	Yes	Yes	Yes	No	Yes	Yes	Yes	Yes	Yes
[59]	Yes	Yes	Yes	No	No	Yes	No	No	Yes
[60]	Yes	No	No	No	Yes	Yes	No	No	Yes
[61]	Yes	Yes	Yes	No	Yes	Yes	Yes	Yes	Yes
[62]	Yes	Yes	Yes	No	Yes	Yes	Yes	Yes	Yes
[63]	Yes	No	No	No	No	No	No	No	Yes
[64]	Yes	Yes	Yes	No	No	Yes	Yes	No	Yes
[65]	Yes	Yes	Yes	No	No	Yes	No	No	Yes
[66]	Yes	Yes	Yes	No	No	Yes	No	No	Yes
[67]	yes	No	No	No	Yes	Yes	No	No	Yes
[68]	Yes	Yes	Yes	No	Yes	Yes	No	No	Yes
[69]	Yes	Yes	Yes	No	Yes	Yes	No	No	Yes
[70]	Yes	Yes	Yes	No	Yes	Yes	No	No	Yes
[71]	Yes	Yes	Yes	No	Yes	Yes	No	No	Yes
[72]	Yes	Yes	Yes	No	Yes	Yes	No	Yes	Yes
[73]	Yes	Yes	Yes	Yes	Yes	Yes	No	Yes	Yes
[74]	Yes	Yes	Yes	Yes	Yes	Yes	No	No	Yes
[75]	Yes	Yes	Yes	No	Yes	Yes	No	Yes	Yes
[76]	Yes	Yes	Yes	No	Yes	Yes	No	No	Yes
[56]	Yes	No	No	No	Yes	Yes	No	No	Yes
[57]	Yes	Yes	Yes	No	Yes	Yes	No	No	Yes
[58]	Yes	Yes	Yes	No	Yes	Yes	Yes	Yes	Yes
[59]	Yes	Yes	Yes	No	No	Yes	No	No	Yes

**Table 3:** Main bioactive compounds of *Bacopa monieri* L. and its biological actions.

Ref.	Bioactive Compounds	Molecular Structures	Health Effects
[88,89]	Asiatic acid	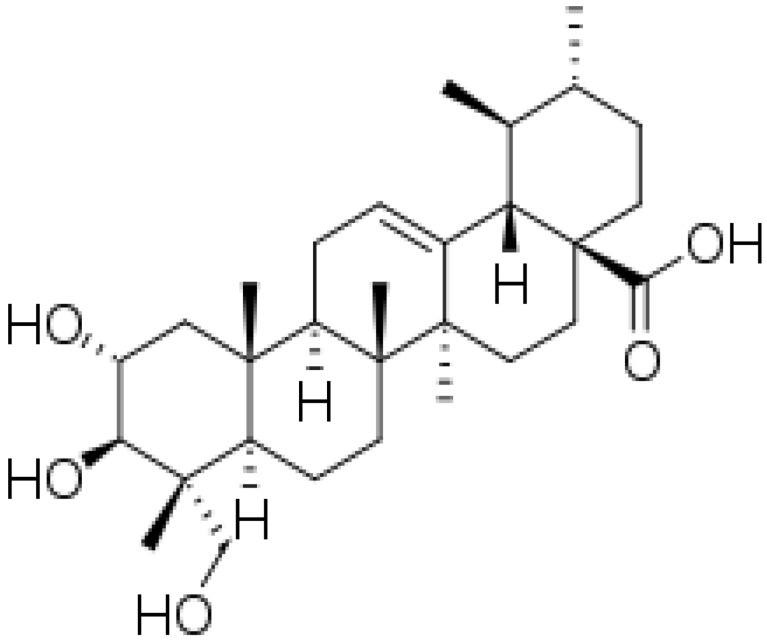	Anti-inflammatory, antioxidant, anti-infective, antitumor, antimicrobial, neuroprotective, diuretic, and immunostimulatory
[90,91]	Aspartic acid	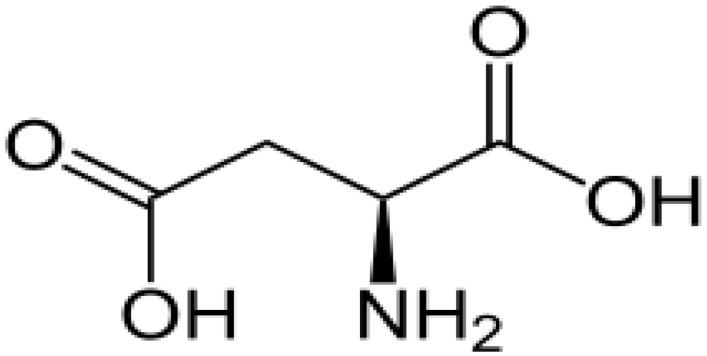	Anti-inflammatory, antioxidant, anticancer, antibacterial, and fundamental role in memory
[92]	β-sitosterol	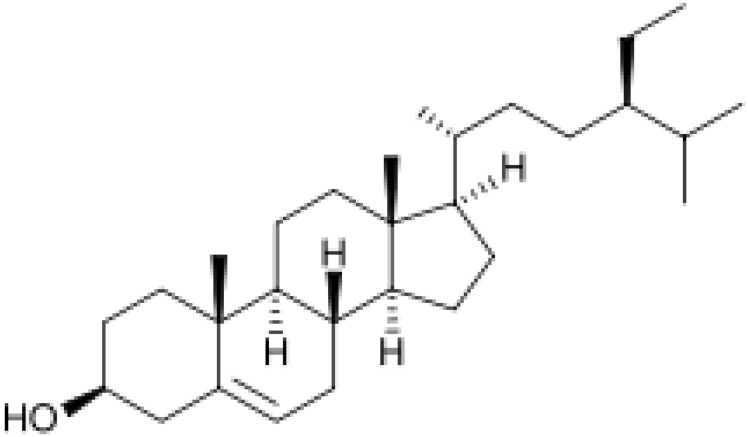	Anti-inflammatory, anticancer, hepatoprotective, antioxidant, cardioprotective, antidiabetic, anti-apoptotic and antihistamine
[40,93]	Bacogenin	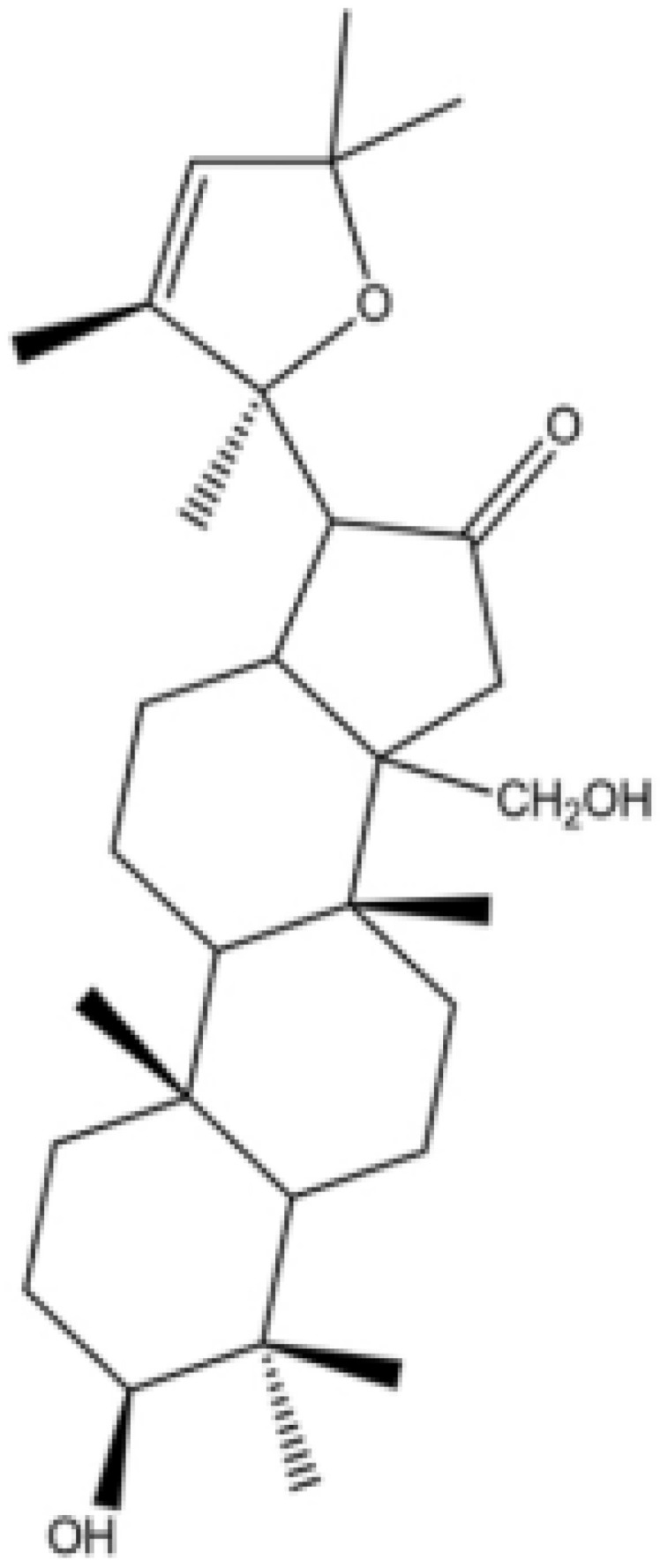	Anti-inflammatory and antioxidant
[45]	Bacopasaponin C	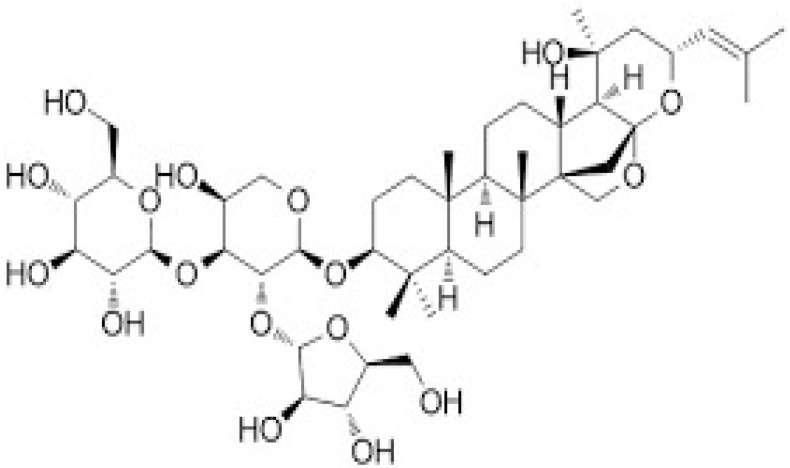	Improvement of neurogenesis and cerebral blood flow, antioxidant, and anti-inflammatory
[83,94]	Bacopaside I	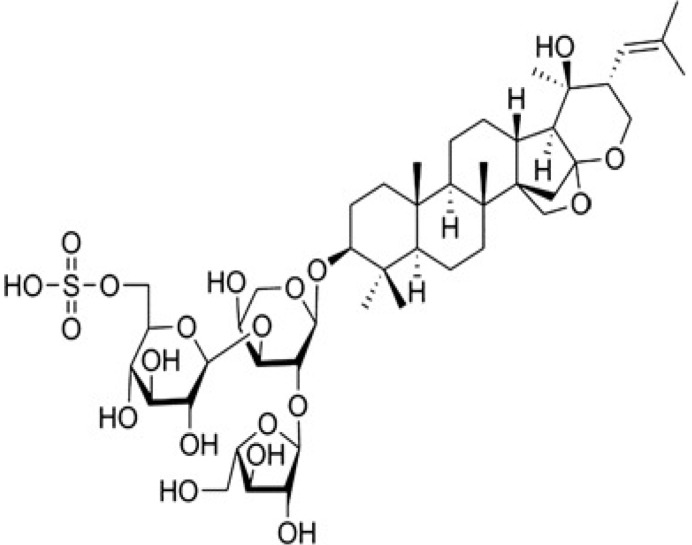	Anti-inflammatory, antioxidant, anti-apoptotic, antidepressant, anticancer, and antiseizure
[84,94]	Bacopaside II	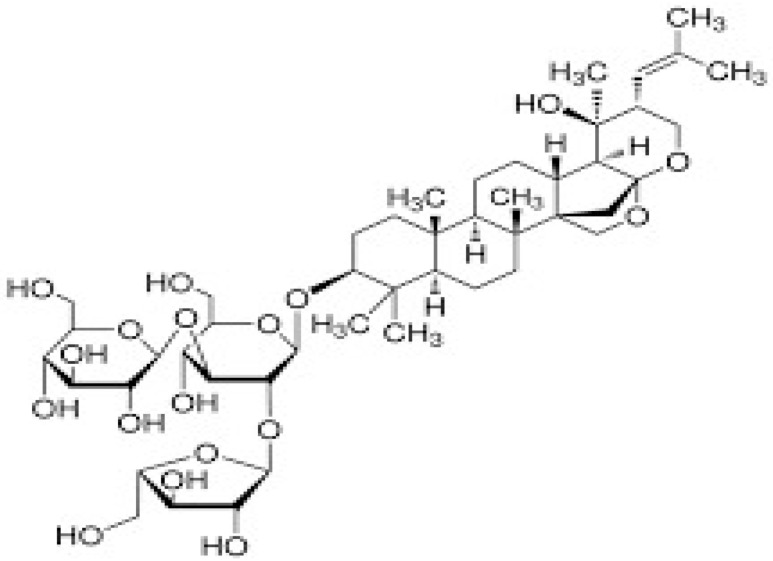	Anti-inflammatory, antioxidant, anticancer, anti-apoptotic, and neuroprotective
[45,94,95]	Bacoside A	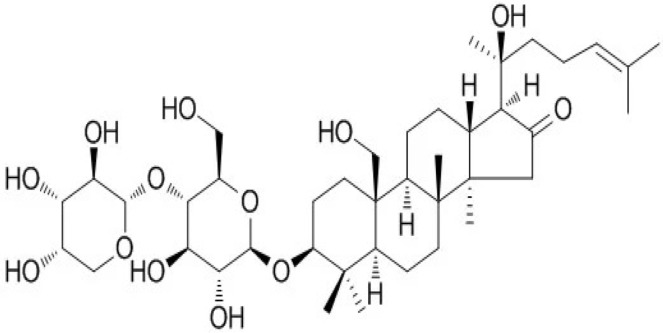	Anti-inflammatory, antioxidant, antiseizure, anti-apoptotic, neuroprotector, and anti-epileptic
[45,95]	Bacoside B	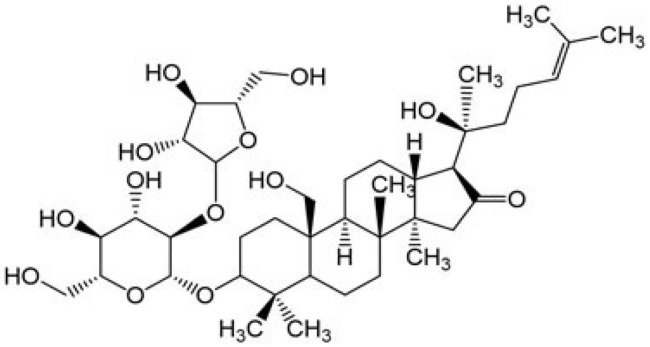	Anti-inflammatory and antioxidant,
[96,97]	Bacosine	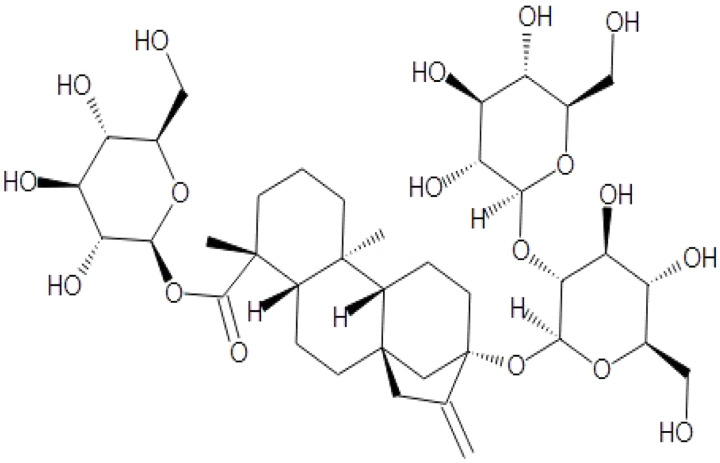	Anti-inflammatory, antioxidant, anticancer, antitumor, antihyperglycemic and hepatoprotective
[45,98,99]	Betulinic acid	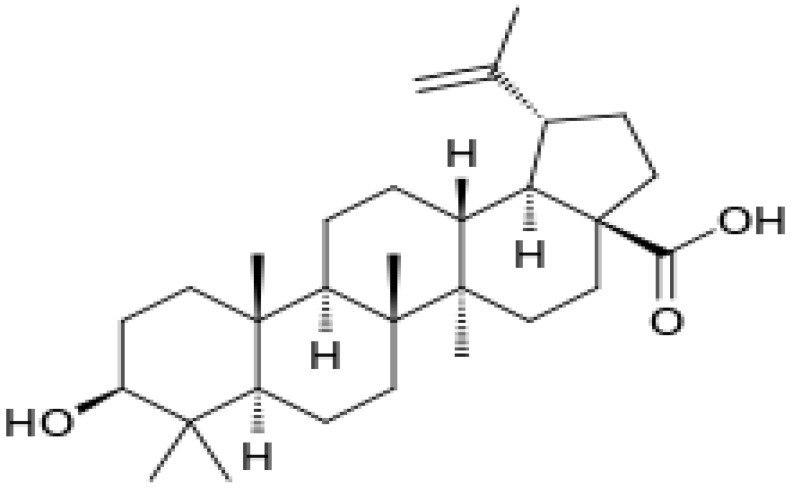	Anti-inflammatory, anticancer, antibacterial, antiviral, antidiabetic, antimalarial, anti-HIV, and antitumor
[100]	Cucurbitacin B	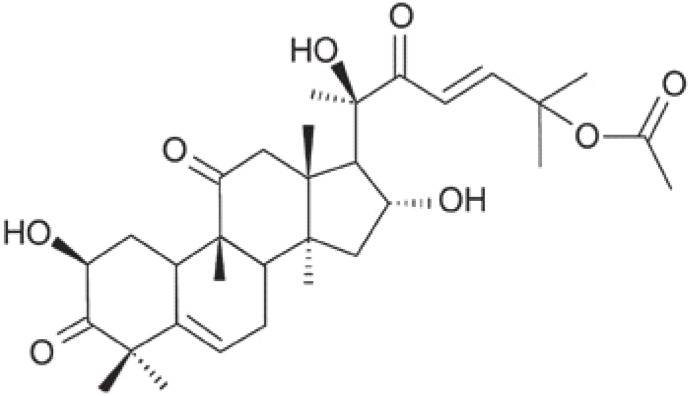	Anti-inflammatory, antioxidant, antiviral, hypoglycemic, hepatoprotective, neuroprotective, and anticancer
[101]	D-mannitol	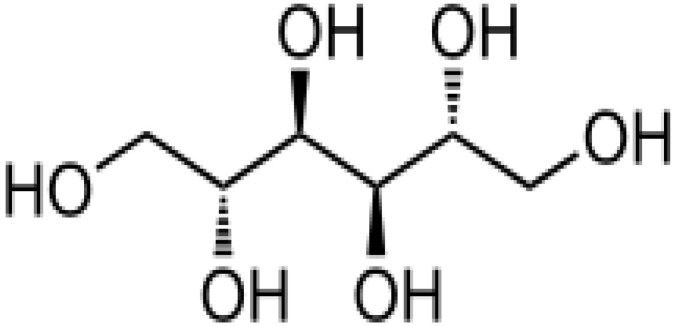	Antitumor and immune stimulants
[102,103]	Glutamic acid	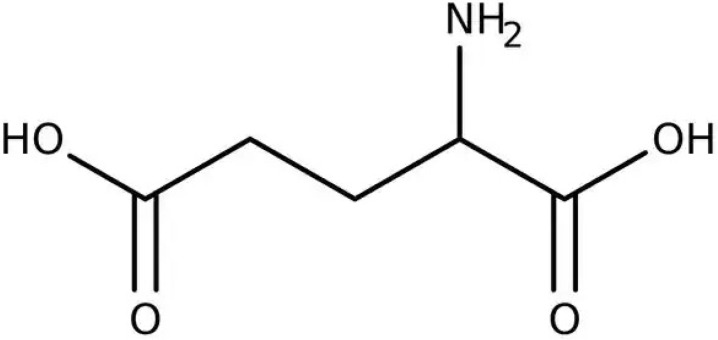	Anticancer, antimicrobial, nephroprotection, and antidiabetic
[37,45]	Jujubogenin	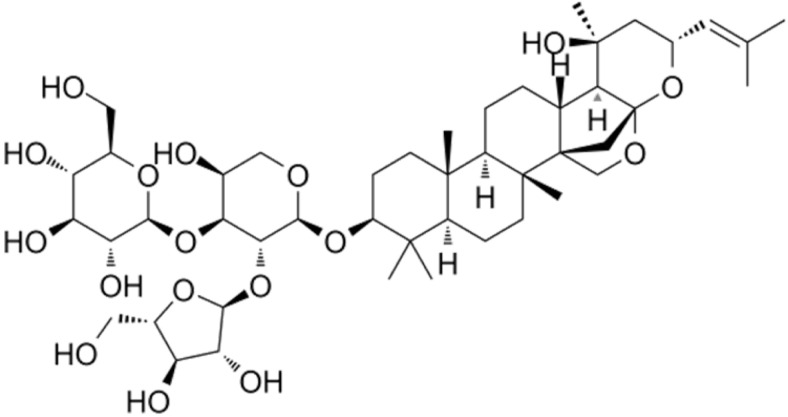	Anti-inflammatory and antioxidant
[104,105]	Loliolide	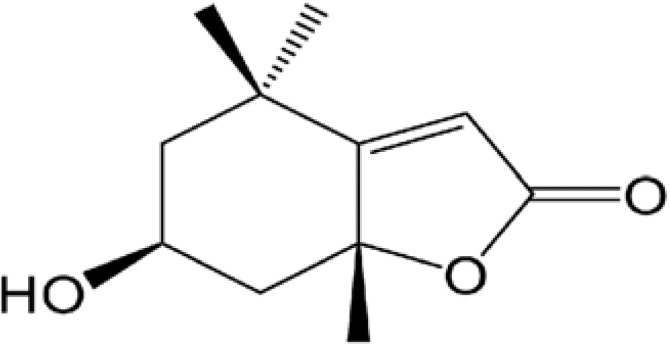	Anti-aging, antioxidant, anti-inflammatory, neuroprotector, anti-Parkinson, anti-cholinesterase and antidepressant
[37,106]	Pseudojujubogenin glycoside	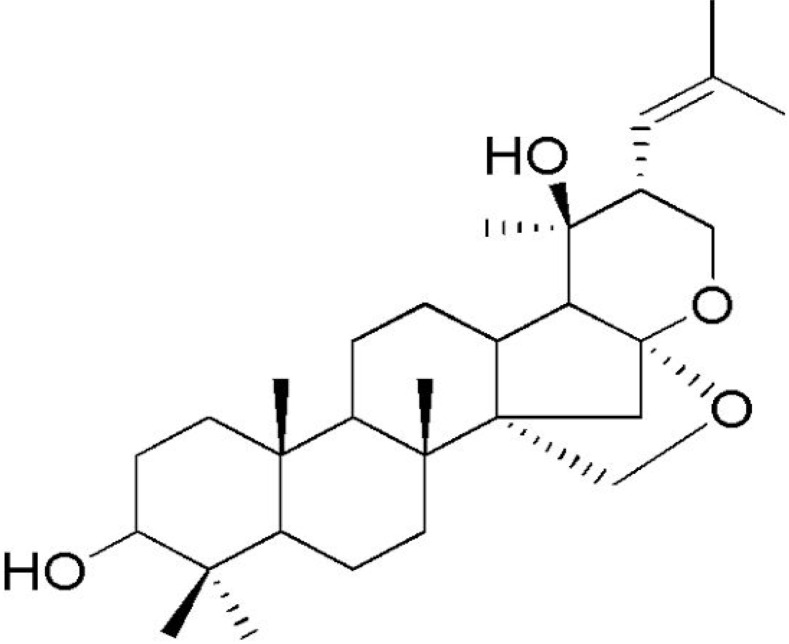	Anti-ageing, anticancer, anticonvulsant, antidepressant, anti-emetic, anti-inflammatory. and antibacterial
[107,108]	Quercetin	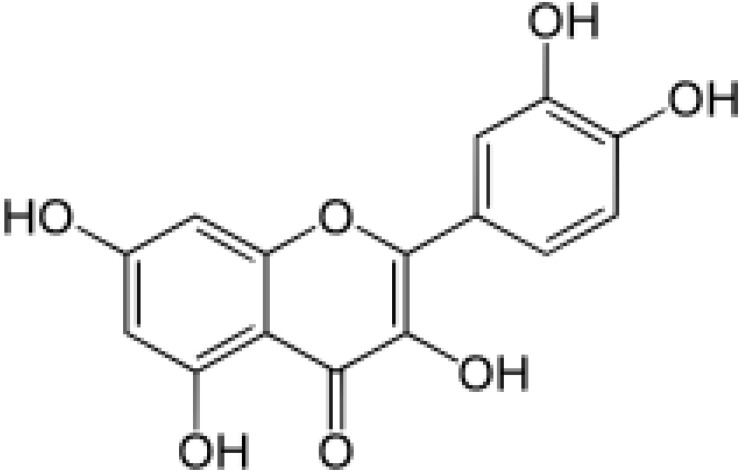	Antioxidative, anti-inflammatory, anti-proliferative, anti-carcinogenic, antidiabetic, and antiviral
[109,110]	Stigmastanol	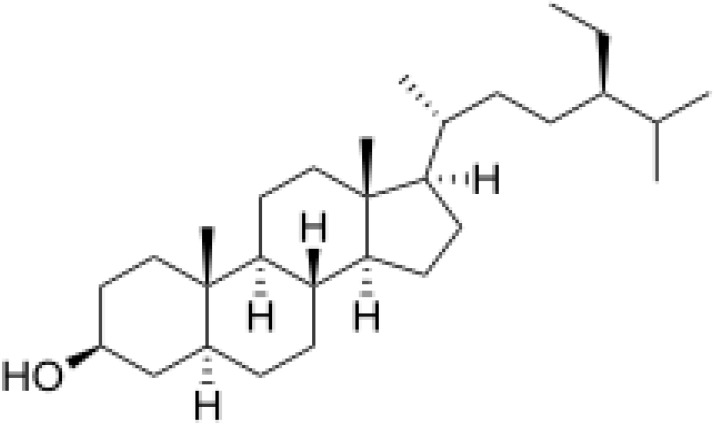	Acaricidal effects and antioxidant
[111]	Stigmasterol	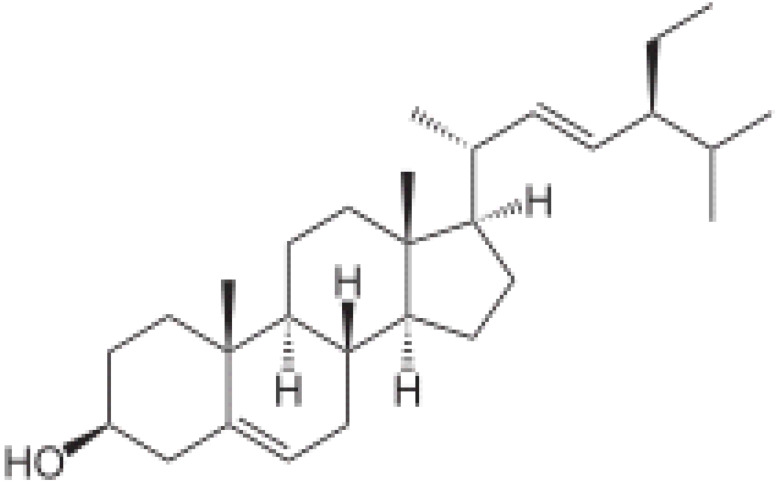	Anticancer, anti-osteoarthritis, anti-inflammatory, antidiabetic, immunomodulatory, antiparasitic, antifungal, antibacterial, antioxidant, and neuroprotective

## Abbreviations

Aβ42, amyloid-β42; ACTH, corticotrophic hormone; ADAS-Cog, Alzheimer’s disease assessment scale-cognitive subscale; ADHD, attention-deficit hyperactivity disorder; AMPA, α-amino-3-hydroxy-5-methyl-4-isoxazolepropionic acid; AQP, aquaporin; ASD, autism spectrum disorder; AVLT, Rey Auditory Verbal Learning Test; BACE, beta-site amyloid precursor protein cleaving enzyme; Bax, Bcl-2-associated X protein; Bcl, B-cell lymphoma; BDNF, Brain-derived neurotrophic factor; BM, Bacopa monnieri L.; BME, Bacopa monnieri ethanolic extract; CAT, catalase; CCR, C-C chemokine receptor; ChAT, Choline Acetyltransferase; CNS, Central Nervous System; COX, cyclooxygenase; CREB, Cyclic AMP response element-binding protein; DAMP, damage-associated molecular patterns; DCX, Doublecortin neuronal marker; DM2, type 2 diabetes mellitus; DSM-IV, Diagnostic and Statistical Manual of Mental Disorders; ERK, extra-cellular signal-regulated kinase; FADD, FAZ-associated death domain protein; GCLC, glutamate cysteine ligase catalytic subunit; GDS, geriatric depression scale; GPx, Glutathione peroxidase; GSH, Glutathione; GSK-3B, glycogen synthase kinase; GST, glutathione S-transferase; GWI, Gulf War Illness; HO, heme oxygenase; ICF, chronic liver failure; IKK, The inhibitor of nuclear factor-κB kinase; IL, Interleukin; INF- γ, Interferon-γ; iNOS, inducible Nitric Oxide synthase; JNK, c-Jun N-terminal kinases; LOX, Lipoxygenase; LPS, lipopolysaccharide; MAO, mono-amino oxidase; MARK, Microtubule affinity regulating kinase; MCP, Monocyte chemoattractant protein; MDA, Malonaldehyde; MMP, Matrix Metalloproteinase; MMSE, Mini-Mental State Examination; MoCA, Montreal Cognitive Assessment; MPTP, 1-methyl-4-phenyl-1,2,3,6 tetrahydropyridine; MTF, multitasking framework; NF-κB, Nuclear Factor-κB; NMDAR, N-methyl-D-aspartate receptors; nNOS, neuronal nitric oxide synthase; NO, Nitric Oxide; 3-NPA, 3-nitropropionic acid; Nrf2, Nuclear factor erythroid 2-related factor 2; OGD, oxygen and glucose-deprivation; OS, oxidative stress; PGES, Prostaglandin; PGIMS, postgraduate institute memory scale; PGQL, Parkinson’s Disease Quality-of-Life; PPI, protein-protein interactions; PSQ, Perceived Stress Questionnaire; PTUPB, dual inhibitor of COX and epoxide hydrolase; RNS, reactive nitrogen species; ROS, reactive oxygen species; SDAT, Senile Dementia of Alzheimer’s Type; SDQ, strength and difficulties questionnaire; SHAPS, Snaith–Hamilton Pleasure Scale; sHE, soluble Epoxide Hydrolase; SOD, Superoxide Dismutase; STAT, signal transducers and activators of transcription; TAA, thioacetamide; TNF-α, Tumor necrosis factor alpha; TRADD, TNFR1-associated death domain protein; WS, *Withania somnifera* (L.) Dunal.
